# New insights on the anatomy and ontogeny of the largest extinct freshwater turtles

**DOI:** 10.1016/j.heliyon.2021.e08591

**Published:** 2021-12-27

**Authors:** Edwin-Alberto Cadena, Andrés Link, Siobhán B. Cooke, Laura K. Stroik, Andrés F. Vanegas, Melissa Tallman

**Affiliations:** a Universidad del Rosario, Facultad de Ciencias Naturales, Grupo de Investigación Paleontología Neotropical Tradicional y Molecular (PaleoNeo), Bogotá, Colombia; b Smithsonian Tropical Research Institute, Panamá, Panama; c Departamento de Ciencias Biológicas, Universidad de Los Andes, Bogotá, Colombia; d Center for Functional Anatomy and Evolution, Johns Hopkins University School of Medicine, Baltimore, MD, USA; e Department of Biomedical Sciences, Grand Valley State University, Allendale, MI, USA; f Museo de Historia Natural la Tatacoa, La Victoria, Huila, Colombia

**Keywords:** Giant turtles, Colombia, Miocene, Paleobiology, South America, Tatacoa

## Abstract

There are many questions regarding the largest freshwater turtle that ever existed, including how its morphology changed during its ontogeny and how a single ecosystem was able to support more than one group of giant turtles. Here, we report the first individual preserving an associated skull and shell for *Stupendemys geographica* (currently the largest known side-necked turtle) and a nearly complete skull of *Caninemys tridentata* found in Miocene rocks of the Tatacoa Desert in Colombia. These two specimens indicate that more than two large freshwater turtle species shared a single ecosystem during the middle Miocene in northern South America. We also show the changes in the shell and scutes that occurred along the ontogeny of *S. geographica*, including a flattening of the carapace, constriction of the vertebral scutes, and increase in the height and thickness of the nuchal upturn wall; some of these changes are also evident in extant representatives of *Podocnemididae*, and have implications for a better understanding of their phylogeny.

## Graphical abstract

**Figure:**
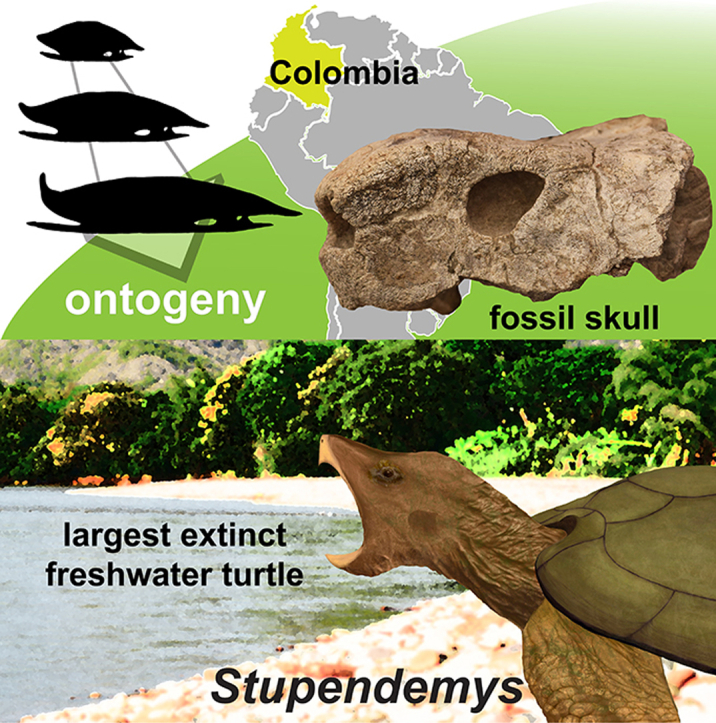

## 1 Introduction

Fossils of giant extinct turtles are known in Cenozoic fluvial and fluvio-deltaic rock sequences in northern South America (Williams, 1956; Wood, 1976; Meylan et al., 2009; Cadena et al., 2012, 2020a). Recently, a study of new fossils of the largest of these giant turtles, *Stupendemys geographicus* (Wood, 1976), has contributed to a better understanding of its anatomy, including morphological expressions of sexual dimorphism (Cadena et al., 2020a). However, other aspects of its paleobiology and paleoecology, such as ontogenetic morphological changes and the ability of the ecosystem it inhabited to support other contemporaneous large turtles have not yet been resolved.

Ontogenetic development in vertebrates can produce drastic changes in skeletal morphology, a key aspect to consider in extinct taxa to avoid incorrect taxonomic identifications, overestimation of paleobiodiversity, or erroneous paleobiological interpretations, among other issues. Fossil vertebrates for which ontogenetic changes have been widely documented include dinosaurs and mammals, with reports of dietary shifts, changes in bone histology, and marked cranial modifications (e.g., Hone et al., 2016; LeBlanc et al., 2016; Sánchez-Villagra 2010; Skawinski and Talanda, 2014; Wang et al., 2017). Among turtles, ontogenetic changes in fossils have been documented in Mesozoic and Cenozoic taxa, including modifications of the anterior scutes of the carapace in *Proterochesis* spp. (Szczygielski et al., 2018); progressive skull stretching and enlargement of posterior and lateral emargination in the Late Cretaceous *Bauremys elegans* from Brazil (Mariani and Romano 2017); and reduction of keels in the neural series of the Eocene *Neochelys franzeni* from Germany (Cadena 2015). None of these taxa represent giant turtles (the largest-bodied species in their ecological category or clade, following Vermeij, 2016).

*Stupendemys geographica* (previously known as *geographicus,* correct spelling emended herein) inhabited northern South America from the middle to the late Miocene (13.5–7 Ma) (Cadena et al., 2020a). So far, all fossil specimens described for this taxon correspond to large shells (full adults) with carapaces longer than 1 m, and reaching a maximum of 2.86 m (both males and females) (Wood 1976; Cadena et al., 2020a). In the most recent study of this taxon, the isolated skull of *Caninemys tridentata* (Meylan et al., 2009) from the late Miocene of Brazil was attributed to *S. geographica* based on the potential match between this skull and the anatomy of lower jaws from la Tatacoa (Colombia) and Urumaco (Venezuela), found in horizons where undisputed shells of *S. geographica* were also discovered (Cadena et al., 2020a). Here, we review this claim, and in light of new fossil specimens described here, reestablish the validity of *C*. *tridentata*. These new fossils correspond to a specimen of an earlier ontogenetic stage, which we attribute to a juvenile or early adult of *S. geographica*, represented by a nearly complete skull, shell, and some postcranial remains; a partial carapace from an adult specimen of *S. geographica*; and a new nearly complete skull from *C. tridentata*, representing the first record of this taxon outside Brazil. We recently discovered these fossils in the north part of the Tatacoa Desert, Huila Department, Colombia, in middle Miocene rocks of the La Victoria Formation (Cadena et al., 2020b). In addition to describing the anatomy of the first skull-shell individual of *S. geographica*, we discuss the ontogenetic changes in the shell of this taxon and extant representatives of *Podocnemididae*, with implications for future fossil identifications of the group. We also present some insights into the paleoecology of the two large coexisting Miocene freshwater *S. geographica* and *C. tridentata* in northern South America.

## 2 Materials and methods

### 2.1 Fossils and extant specimens

We found and prepared the fossil turtles described here (VPPLT-1337, VPPLT-1719, and VPPLT-1720) between 2018 to 2020. All specimens are housed at the Paleontological collection of VPPLT. We used specimens of the extant species *Peltocephalus dumerilianus, Erymnochelys madagascariensis,* and *Podocnemis* spp., as well as those described in Cadena et al. (2020a) for comparisons with the fossil specimens (Supplementary File S1).

### 2.2 Computed tomography (CT)

We scanned the skull of *Stupendemys geographica* specimen VPPLT-1337 using Toshiba Aquilion CT at the Méderi Hospital X-ray facility, Bogotá, Colombia, under the following settings: zoom factor 1.2, 12 902 total images, 120 kV, 150 mA, and 2 mm inter-slice spacing. We analyzed the CT data using iQ-VIEW software (https://www.imagesystems.biz). We also scanned skulls from the extant podocnemidids *Peltocephalus dumerilianus* SMF-40169*, Erymnochelys madagascariensis* SMF-33056*,* and *Podocnemis unifilis* SMF-32915 using a Tomoscope HV 500 (Werth Messtechnik GmbH) with a 2k detector and a 225-kV μ-focus X-ray source in the industrial μCT facility at the Technical University in Deggendorf, Deggendorf, Germany. Scan parameters: CT mode 2, 150 μA, 190 kV, 1200 steps, voxel resolution 20.2 μm). CT data is available in (Supplementary Videos S1–S3). CT data for the extant taxa are available via direct contact to E-A. Cadena, as they are a part of a different research project currently in progress.

Supplementary content related to this article has been published online at https://doi.org/10.1016/j.heliyon.2021.e08591.

The following are the supplementary data related to this article:

Supplementary_VideoS1.MOV Supplementary_VideoS1.MOV

Supplementary_VideoS2.mov Supplementary_VideoS2.mov

Supplementary_VideoS3.mov Supplementary_VideoS3.mov

Supplementary_VideoS4.mov Supplementary_VideoS4.mov

### 2.3 Geometric morphometrics (GM)

#### 2.3.1 Vertebral scutes

The arrangement of contacts and size of the carapace vertebral scutes have been traditionally used in the diagnosis of some fossil taxa and also expressed in several characters included in phylogenetic analyses (see characters 154–159 in Cadena (2015); 203–206 and 207–213 in Hermanson et al. (2020)). In order to explore the potential morphological variability of the vertebral scutes series along the ontogeny of extant and some fossil podocnemidids including *Stupendemys geographica,* we examined 39 specimens including hatchlings (nine specimens), juveniles (11 specimens), and adults (19 specimens). We identified 24 landmarks for the vertebral scutes. Most of these landmarks have been defined and used in previous studies (Caude et al., 2003; Fish and Stayton, 2014). First, we collected the landmark coordinates based on photos of the specimens using the PointPicker plugin in ImageJ 1.52q (Schneider et al., 2012) (Supplementary File S2). Then, we used MorphoJ 1.07a (Klingenberg, 2011) to compute a generalized Procrustes analysis. The Procrustes aligned coordinates were subsequently used in principal components analyses (PCA). We compared the morphology of each of the three *S. geographica* specimens included (VPPLT-1337 described herein, MCZ-4376 and CIAAP-2002-01 described in Wood (1976) and Cadena et al. (2020a)) to the other extant and fossil podocnemidids, adjusting the scaling factor accordingly.

#### 2.3.2 Skull snout

Sixteen two-dimensional skull landmarks were collected on a sample of 32 turtles, encompassing ten different species within the *Podocnemididae* (extant and fossils), including one specimens of *S. geographica* (VPLT 1337) and two specimens of *C. tridentata* (MCZ-4376 and CIAAP-2002-01). Landmarks were taken from pictures in lateral view using the PointPicker plugin in ImageJ 1.52q (Schneider et al., 2012). These landmarks were subsequently analyzed using MorphoJ v.1.07 (Klingenberg, 2011) and the *geomorph* plug in for R v. 4.0 (Adams et al., 2021) (Supplementary File S2). These data were aligned using a generalized Procrustes analysis and subsequent multivariate statistics were performed on these Procrustes aligned coordinates. First, a principal components analysis was used to explore the shape variation in the extant taxa. Using the data from the PCA of the extant individuals, fossil taxa were then “projected” into the PCA space post hoc in order to see how the shape of the fossils relates to that of the extant taxa. Next, the phylogenetic tree built via the parameters in this study (see below) was pruned to contain only branches with corresponding morphometric data using Mesquite v3.7 (Maddison and Maddison, 2021). The morphometric data were then mapped onto the phylogeny to determine whether there is a significant phylogenetic signal in the landmark data. Shapes were calculated for each node in the tree by taking the average coordinates weighted by the inverse of the distance of each individual configuration to each node. In order to visualize directions of evolutionary change, a PCA of the mean shapes was calculated, the shapes of the nodes were projected into the PCA space post-hoc, and the tree branches were drawn, creating a phylomorphospace plot (Klingenberg and Ekau, 1996).

### 2.4 Phylogenetic analysis

For the phylogenetic analysis, we used the matrix of Joyce et al. (2021, references therein), added missing information, re-coded some characters to correct miscoding and some comments on character modifications (Supplementary File S3). The coding for *Stupendemys geographica* was based on the new specimens described here (VPPLT-1337 and VPPLT-1719) and Cadena et al. (2020a). The coding for *Caninemys tridentata* was based on the new skull and shell fragments VPPTL-1720 described here, and the specimen described in Meylan et al. (2009). The new character-taxon matrix is composed of 104 taxa and 268 characters (Supplementary File S4). We performed two different phylogenetic analyses in TNT 1.5 (Goloboff and Catalano 2016) to establish the position of *S. geographica* inside Pan-Pleurodira, using *Proganochelys quenstedti* as the outgroup taxon. The first analysis included all taxa, and the second was focused on the Podocnemidoidae clade, removing all other taxa except *Progranochelys quenstedti*, *Notoemys laticentralis*, and *Platychelys oberndorferi*. We used the following settings for the two analyses: traditional search, 20 trees to save per replication, and other parameters by default; memory tree increased to max. trees 10000; collapse of zero-length branches according to rule 1; light implied weighting *k* value of 12; and 1000 replicates of random addition sequences. All characters were equally weighted and some were treated as ordered (characters 1, 10, 14, 18, 19, 51, 52, 56, 57, 75, 78, 81, 82, 86, 88, 95, 96, 99, 101, 103, 112, 114, 115, 119, 128, 130, 171, 172, 174, 175, 182, 183, 193, 195, 202, 220, 224, 225, 231 and 242, following Joyce et al. (2021)). A strict consensus tree was generated for each of the two analyses and statistics obtained included Consistency (CI) and Retention (RI) indexes and Bremer support, calculated using implemented scripts in TNT. Trees were exported and formatted to circular shape using Fig. Tree v 1.4.4 (http://tree.bio.ed.ac.uk/software/figtree/).

### 2.5 Abbreviations

*Institutional abbreviations*—**CIAAP**, Centro de Investigaciones Antropológicas, Arqueológicas y Palentológicas of the Universidad Nacional Experimental Francisco de Miranda, Coro, Falcón State, Venezuela; **DNPM-MCT**, Departmento Nacional de Produçaõ Mineral, Divisaõ de Geologia e Mineralogia, Ciências da Terra, Rio de Janeiro, Brazil; **MCZ**, Museum of Comparative Zoology, Harvard University, Cambridge, USA; **SMF**, Senckenberg Museum Frankfurt, herpetological collection, Frankfurt, Germany; **VPPLT**, Museo de Historia Natural La Tatacoa, La Victoria, Huila Department, Colombia.

*Anatomical abbreviations***—Abd,** abdominal scute; **ac,** anterior condyle; **acp,** acromial process; **Ana,** anal sute; **ars,** articular surface; **ast,** astragalus; **axb,** axillary buttress; **axn,** axis neural arch; **bm,** bite mark; **bs,** basisphenoid; **bo,** basioccipital; **cal,** calcaneum; **cev,** cervical; **co,** costal; **cor,** coracoid; **dig,** digit; **dta,** distal tarsus; **ent,** entoplastron; **epi,** epiplastron; **Ext,** extragular scute; **Fem,** femoral scute; **fem,** femur; **fpp,** foramen palatinum posterius; **fpt,** fossa pterygoidea; **FR,** frontal scute; **fr,** frontal; **Hum,** humeral scute; **hyo,** hyoplastron; **hyp,** hypoplastron; **Int,** intergular scute; **ju,** jugal; **IP,** interparietal scute; **lp,** lateral process; **M,** marginal scute; **mes,** mesoplastron; **met,** metatarso; **mfo,** musk foramen; **mx,** maxilla; posorbital scute; **nc,** nuchal upturn crest; **ne,** neural; **nu,** nuchal; **op,** opisthotic; **P,** pleural scute; **PA,** parietal scute; **pa,** parietal; **pc,** posterior condyle; **pe,** peripheral; **Pec,** pectoral scute; **pha,** phalange; **pl,** palatine; **pm,** premaxilla; **PO,** postorbital scute; **po,** postorbital; **poz,** postzygapophysis; **pr,** prefrontal; **prz,** postzygapophysis; **pt,** pterygoid; **ptt,** processus trochlearis pterygoidei; **py,** pygal; **qj,** quadratojugal; **qu,** quadrate; **sap**, superior articular process; **so,** supraoccipital; **sp,** suprapygal; **sq,** squamosal; **tcp**, thoracic process; **tv,** thoracic vertebra; **V,** vertebral scute; **vfo**, vertebral foramen; **vo,** vomer; **vsm**, visceral surface margin; **xip,** xiphiplastron.

## 3 Results

### 3.1 Systematic palaeontology

*TESTUDINES* Batsch 1788 (Joyce et al., 2020b)

*PLEURODIRA* Cope 1865 (Joyce et al., 2020a)

*PODOCNEMIDIDAE* Cope 1868 (Joyce et al., 2021)

*ERYMNOCHELYINAE* Broin 1988 (Joyce et al., 2021)

**Figure 1:**
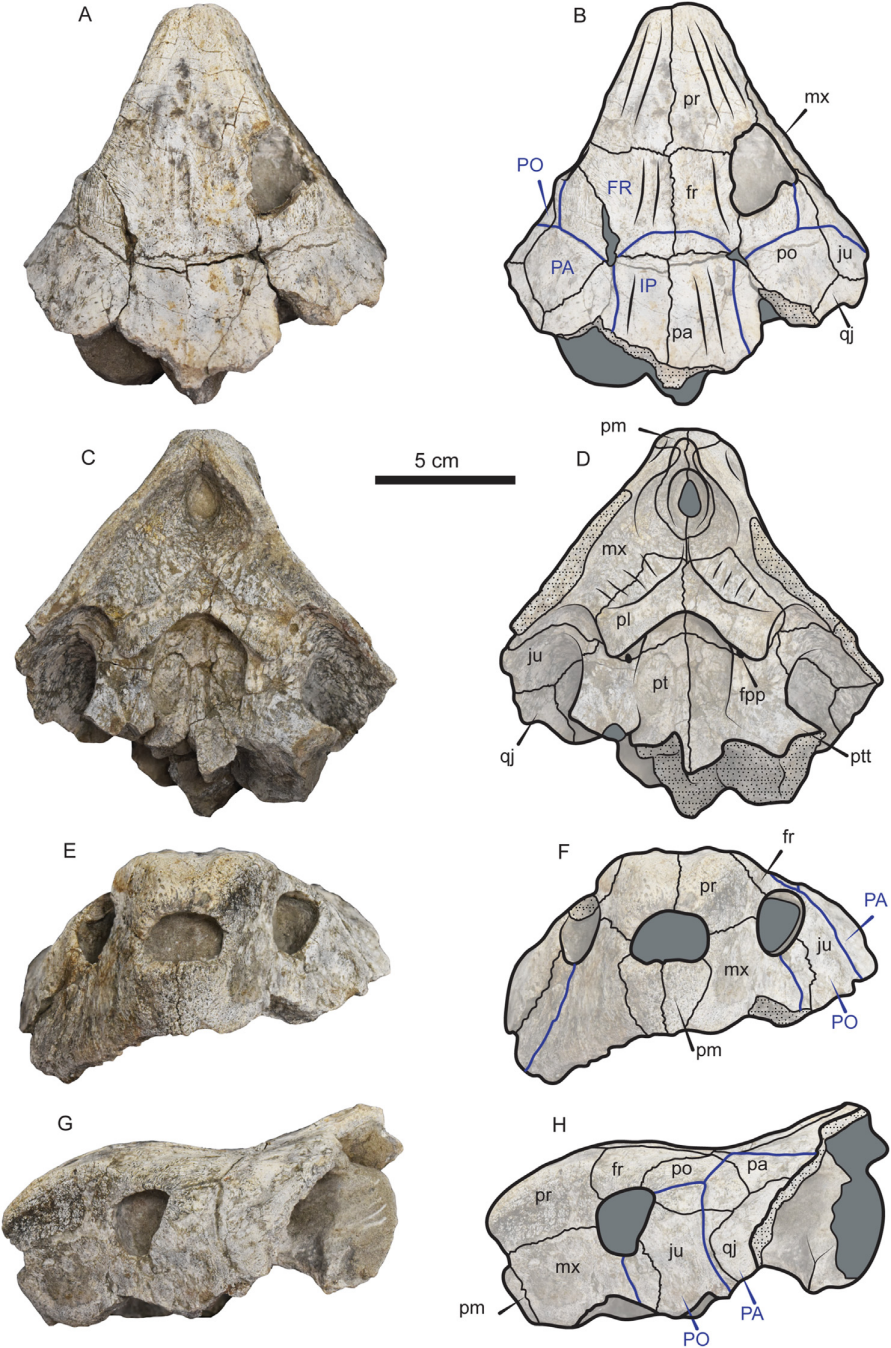
Photographs and line drawings of the skull of *Stupendemys geographica* VPPLT-1337 specimen. (A–B) Dorsal view. (C–D) Ventral view. (E–F) Anterior view. (G–H) Left lateral view. Blue lines indicate scutes. Dark gray shaded indicates rock matrix, and pitted texture indicates broken surfaces of the bones. For abbreviations see Materials and Methods (numeral 2.5.).

**Figure 2:**
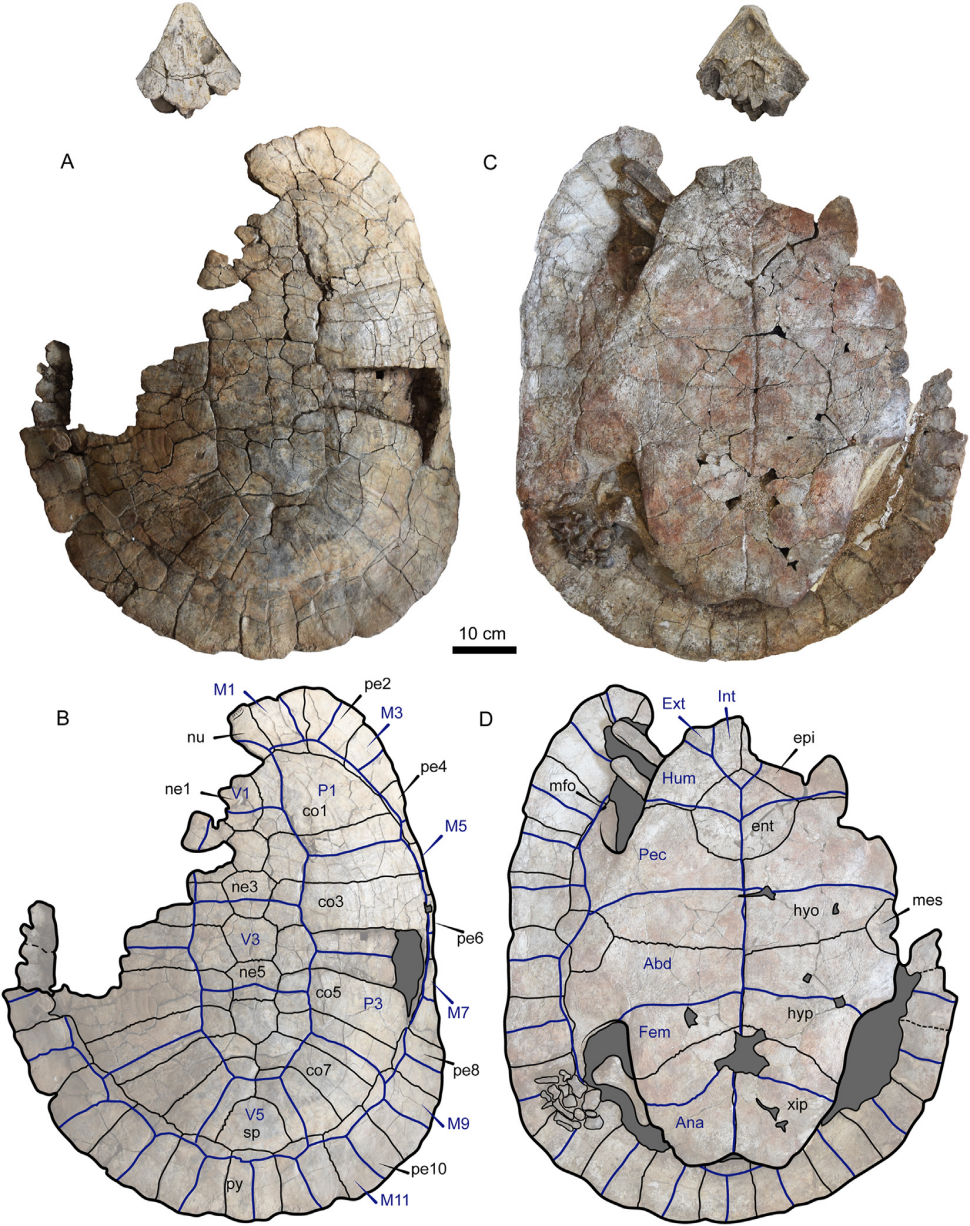
Photographs and line drawings of the shell of *Stupendemys geographica* VPPLT-1337 specimen. (A–B) Dorsal view, together with the skull to show their relative size. (C–D) Ventral view. Blue lines indicate scutes. Dark gray shaded indicates rock matrix. For abbreviations see Materials and Methods (numeral 2.5.).

**Figure 3:**
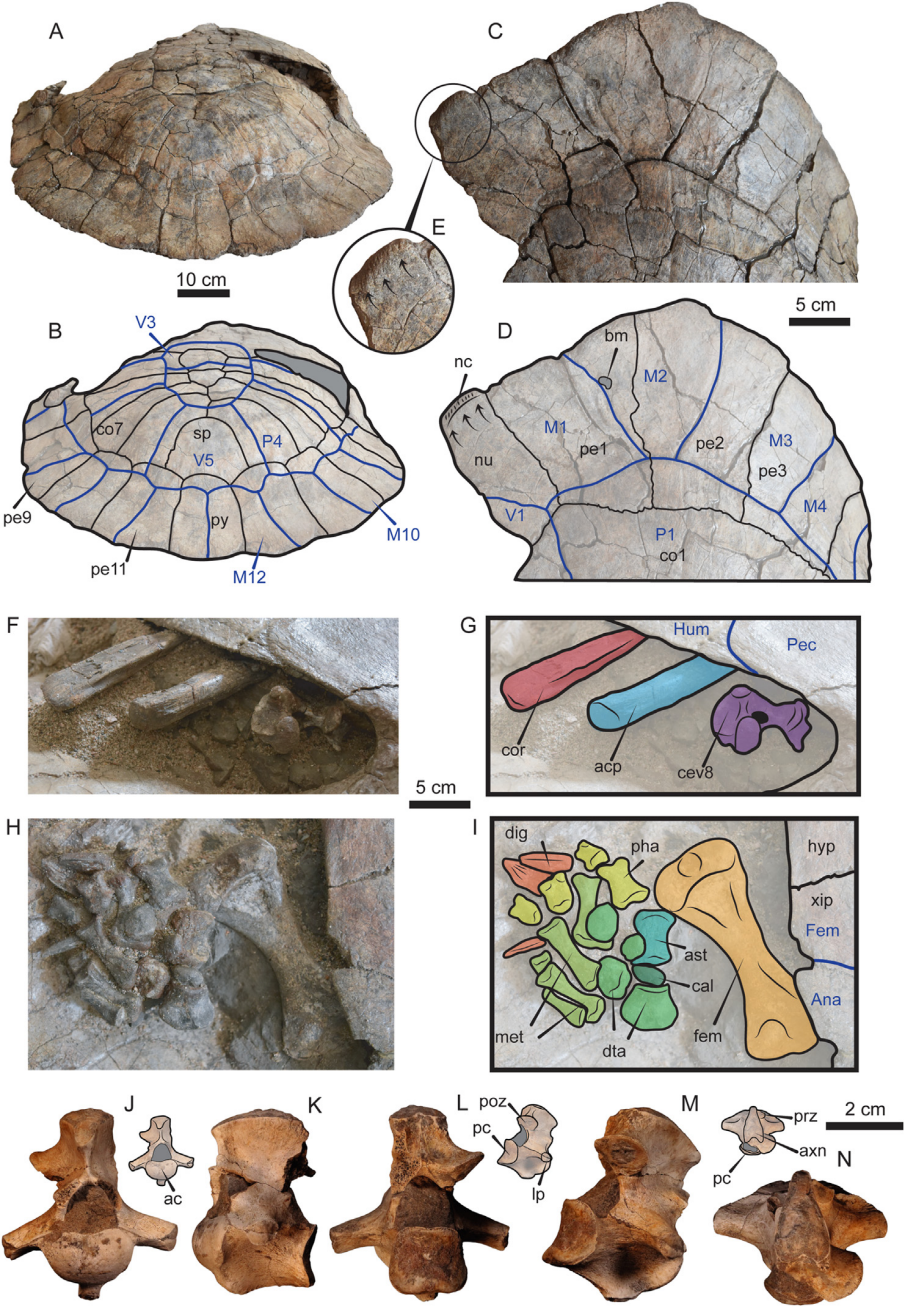
Additional photographs and line drawings of the shell of *Stupendemys geographica* VPPLT-1337 specimen and some of its postcranial bones. (A–B) Carapace in posterodorsal view, scale bar 10 cm. (C–E) Close-up of the right anterior region of the carapace, showing the upturn anterior edge of the nuchal, upper scale bar 5 cm. (F–G) Close-up of the right anterior bridge (hyoplastron-carapace) region, showing the left coracoid, acromion, and cervical 8. Central scale bare 5 cm. (H–I) Close-up of the right posterior bridge (hyoplastron-carapace) region, showing the right femur and most of the pes elements. Central scale bare 5 cm. (J–N) Cervical 2 in anterior view (J), right lateral view (K), posterior view (L), left posterolateral view (M), and dorsal view (N), showing the typical horse-saddle shape of the posterior condyle of podocnemidids. Scale bar 2 cm. Blue lines indicate scutes. Dark gray shaded indicates rock matrix. For abbreviations see Materials and Methods (numeral 2.5.).

**Figure 4:**
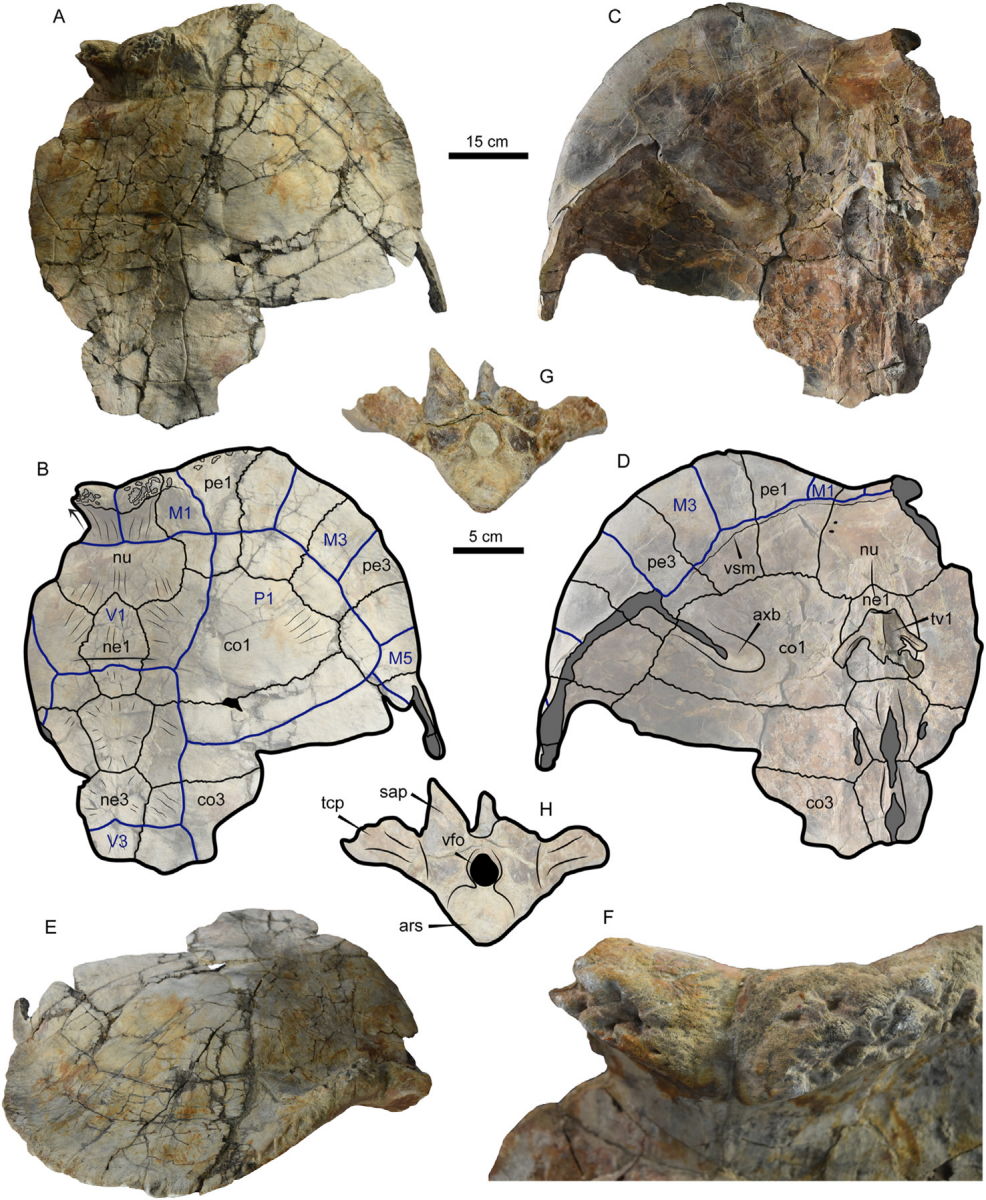
Photographs and line drawing of *Stupendemys geographica* VPPLT-1719 specimen, adult female. (A–B) Carapace in dorsal view. (C–D) Carapace in ventral view. (E) Carapace in anterior view. (F) Close-up of the nuchal showing the thick anterior upturn wall and deep pits. (G–H) Thoracic 1 in anterior view.

*Stupendemys geographica*Wood (1976). (emended spelling) Figures 1, 2, 3, 4, and 12A, Supplementary File S5

*New referred material—*VPPLT-1337 (Figures 1, 2, and 3), represented by a partially preserved skull, articulated shell (carapace and plastron), most of the elements of the right hindlimb, the right pectoral girdle bones, and cervical vertebrae 2 and 8, all from the same individual. VPPLT-1719 (Figure 4, Supplementary File S5), represented by the middle to right anterior portion of a carapace. Measurements for both specimens VPPLT-1337 and VPPLT-1719 are presented in Table 1.

**Table 1:** Measurements for *Stupendemys geographica* and *Caninemys tridentata* specimens described herein, given in cm as they are preserved.

Specimen	CLm	CLp	CWm	PLp	PWm	SL	SW
*Stupendemys geographica*							
VPPLT-1337	86.2	90.1	71.4	68.3	51	15.2	13.1
VPPL-1719	63.4	65.8	77.2				
*Caninemys tridentata*							
VPPLT-1720						20.3	15.6

Abbreviations—CLm, carapace length midline; CLp, carapace length parasagittal; CWm, carapace width midline; SL, maximum skull length; SW, maximum skull width; PLp, plastron length parasagittal; PWm, plastron width midline.

**Figure 5:**
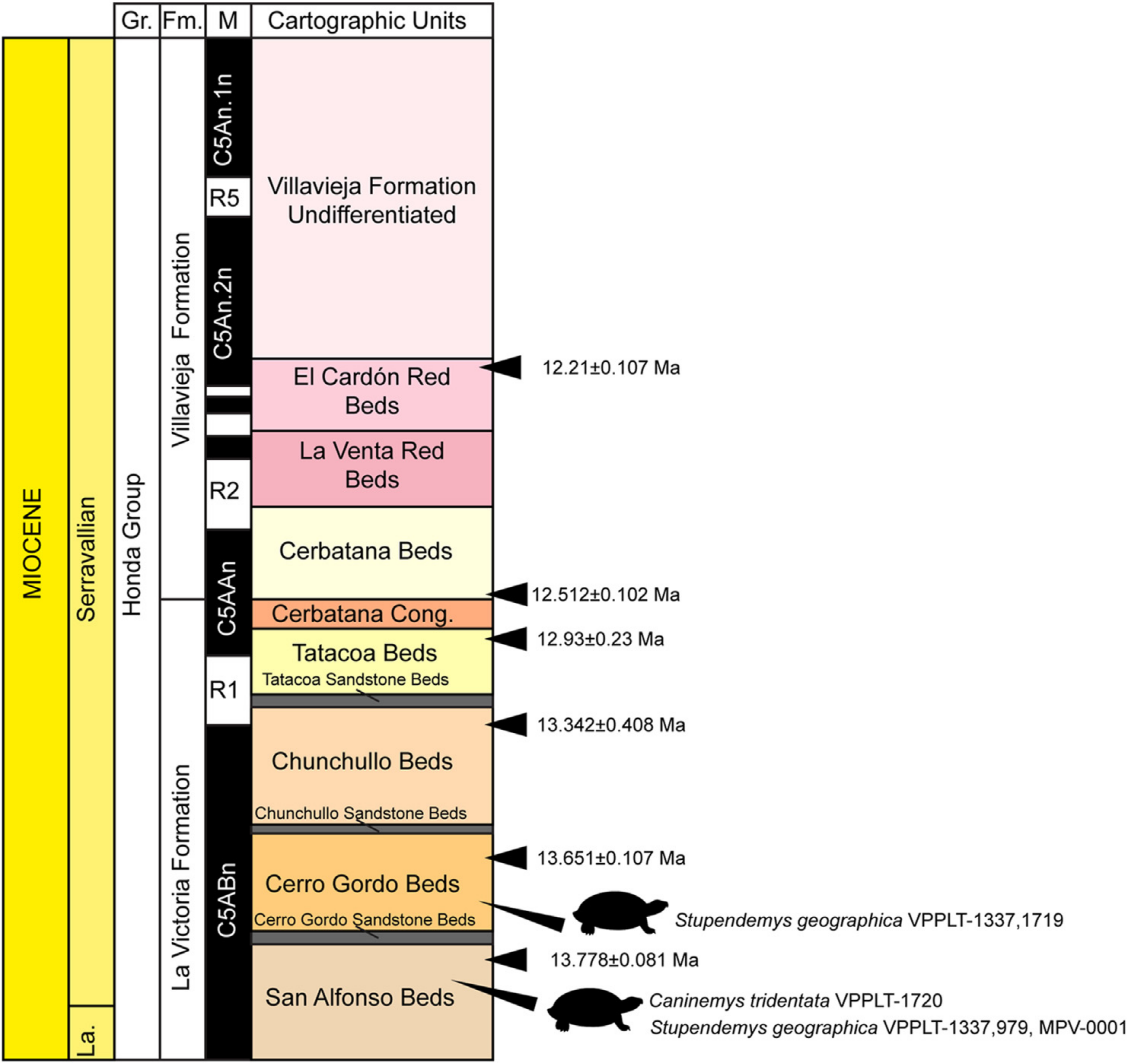
Stratigraphic framework for the Tatacoa Desert, showing the occurrence of *Stupendemys geographica* and *Caninemys tridentata* in the lower segment of La Victoria Formation. Figure taken, modified and redrawn from Montes et al. (2021).

*Localities, stratigraphy and age—*VPPLT-1337 comes from the base of the Cerro Gordo hill locality (3º18′25.05″N, -75º9′6.48″W, elevation 374 m), lower segment of La Victoria Formation (Guerrero, 1997), dated as middle Miocene (13.651 +/− 0.107 Ma) (Montes et al., 2021) (Figure 5). VPPLT-1719 comes from Casezing (Cerro Gordo) locality (3º19′22.72″N, -75º8′57.40″W, elevation 427 m), La Victoria Formation (Guerreo, 1997), Cerro Gordo Beds (Figure 5).

*Revised diagnosis—Stupendemys geographica* is recognized as a pleurodire based on: 1) sutural articulation of pelvis with shell; 2) loss of medial contact of mesoplastra; 3) well-developed anal notch; 4) fusion of gulars; 5) formed central articulations of cervical vertebrae; and 6) a well-developed processus trochlearis pterygoidei. It is a podocnemidid based on: 1) palatine forming moderate amount of the upper triturating surface and 2) a cervical centrum with saddle-shaped posterior condyle. *Stupendemys geographica* differs from all other podocnemidids in each of its anatomical elements as follows. Skull: 1) an upper triturating surface formed by the posteromedial portion of premaxillae, medial contact between maxillae, and palatines forming a secondary palate; 2) an extremely inflated snout formed by prefrontals; 3) anteroventrally descending wall of prefrontals onto the narium externa aperture; 4) striated surface of skull roof bones (prefrontals, frontals, and parietals). Lower jaw: 1) a deep triturating surface, forming an oval concavity; 2) labial ridge curved anteriorly ending in an acute tip; 3) lingual ridge is a blunt margin forming an accessory ridge that increases in height and width anteriorly and runs as a narrow ridge at the medial symphysis; 4) high coronoid process; 5) large dorsal opening of fossa Meckelii, which fills the posterior end of the jaw to such an extent that the area articularis mandibularis forms part of the posterior margin, and the fossa opens posterolaterally next to the jaw articulation. Carapace: 1) low-arched carapace decreasing in height along ontogeny; 2) irregular nodular contours on external surface and deep median notch at front; 3) anterior border of nuchal-peripheral bones thickened and moderately (juvenile-adult) to strongly (adult) upturned; 4) extremely long peripherals 1 and 2 forming massive anterolateral horns slightly projected ventrally in forms attributed as male; 5) carapace dorsal bone surface smooth to striated or slightly pitted; 6) posterior peripheral bones moderately scalloped along margins; 7) vertebral scute 5 being the longest and widest of the series particularly in adult individuals. Plastron: 1) pectoral-abdominal sulcus very anterior to mesoplastra, reaching almost the hyoplastra lateral notch level in adults. Neck: 1) cervical vertebrae (probably 7 and 8) with neural arches relatively high in relation to anteroposterior lengths of centra; 2) articular facets of postzygapophyses forming acute angle of less than 90°; 3) cervical 8 neural arch with large horizontal plane, prezygapophyses directed perpendicularly, thin bladelike spine on anterior face of neural arch and no ventral keel on centrum. Humerus: 1) humerus squat, massive; 2) deep bicipital fossa between lateral and medial articular facets on ventral surface; 3) prominent ridge traversing ventral surface of shaft from medial process to distal end, terminating just above lateral condyle; 4) medial condyle broadest at anterior end; 5) medial and lateral condyles facing very ventrally; 6) straight to slightly slender shaft and more triangular in cross section than circular. Femur: 1) femur squat, massive, greatly flattened dorsoventrally particularly in adults; 2) breadth of tibial condyle approximately one-third total length of bone. Scapula: 1) dorsal strongly bowed scapular process with a flattened flange projecting laterally from the main axis.

It shares with *Peltocephalus dumerilianus, Erymnochelys madagascariensis* and closer relatives: 1) large postorbital bones avoiding a contact between jugals and parietals; 2) cheek emargination extremely reduced to almost absent; 3) anterior overhang of prefrontals completely covering the narium externa aperture ending in a straight to convex edge; 4) laterally facing orbits in dorsal view of the skull, with a very narrow to almost complete absent dorsal exposure of the maxilla and jugal; and 5) an articular with a processus retroarticularis posteroventromedially projected, differing from the ventrally projected *Podocnemis* spp. It shares with *Peltocephalus dumerilianus*: 1) an interparietal scute with lateral margins tapering posteriorly; 2) ventral premaxillae forming a very deep medial concavity (cranial pit); 3) pinched snout; 4) long contact between prefrontal and maxilla in lateral view (orbitonarial bar); 5) vertebral 1 scute nearly square in shape with parallel margins ontogenetically conservative; and 6) axillary buttress reaching peripheral 3.

*Remarks—*The specific epithet ‘*geographicus*’ is incorrect because *Stupendemys* constitutes a feminine generic name. Its associated specific epithets therefore must also be feminine following the International Commission on Zoological Nomenclature (ICZN, 1999:Art. 30.1.2) and Vlachos (2015), and we correct this name to *Stupendemys geographica.* For details on the holotype and other hypodigms of this taxon, see Cadena et al. (2020a) and discussion below (which includes the skull of *Caninemys tridentata,* the holotype of which is described by Meylan et al. (2009)).

*Caninemys tridentata*Meylan et al. (2009) (reestablished taxon)

Figures 6 and 7

**Figure 6:**
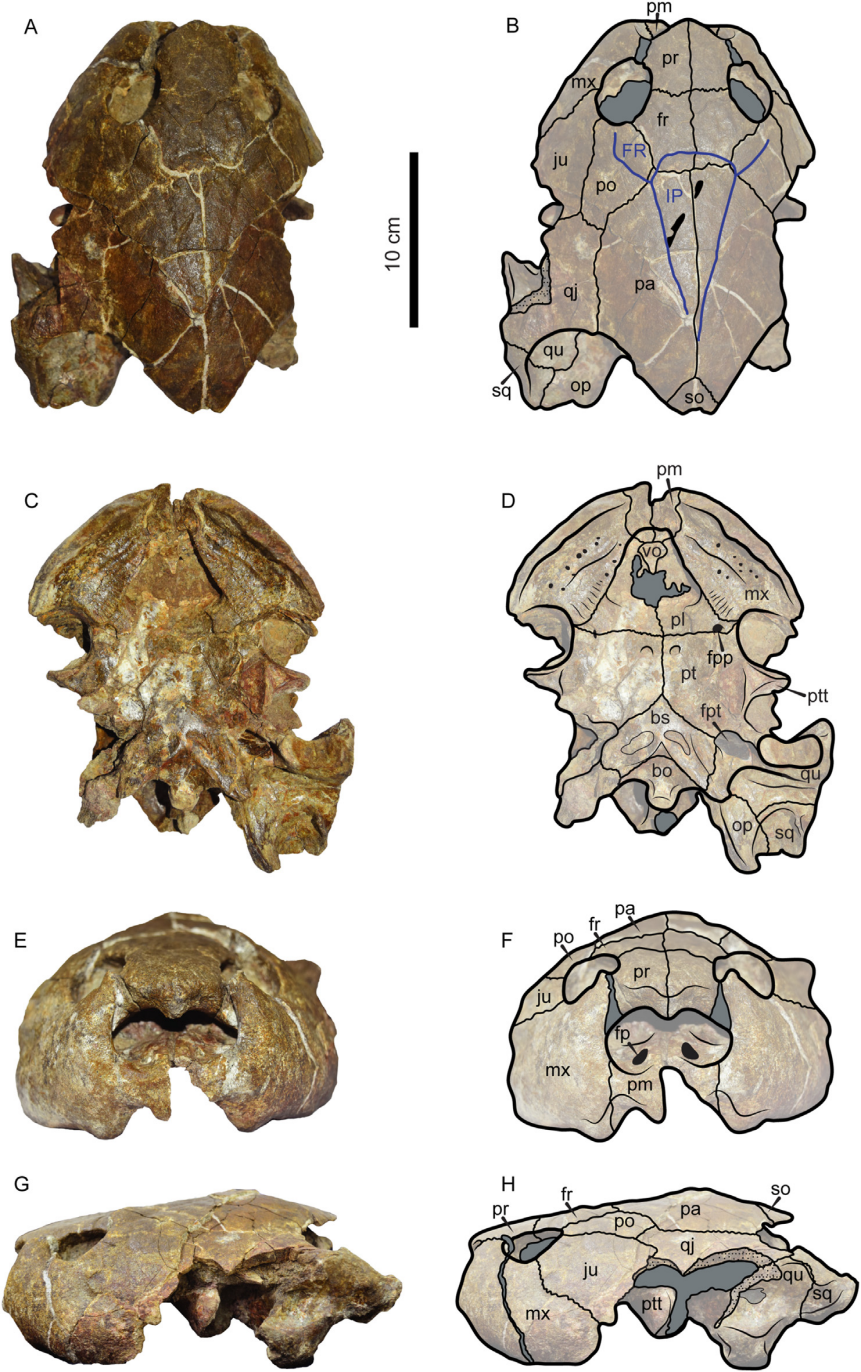
Photographs and line drawings of the skull of *Caninemys tridentata* VPPLT-1720 specimen. (A–B) Dorsal view. (C–D) Ventral view. (E–F) Anterior view. (G–H) Left lateral view. Blue lines indicate scutes. Dark gray shaded indicates rock matrix, pitted texture indicates broken surfaces of the bones, oval to circular black shaded areas indicate foramina or pits, and black shaded irregular areas indicate scars. For abbreviations see Materials and Methods (numeral 2.5.).

**Figure 7:**
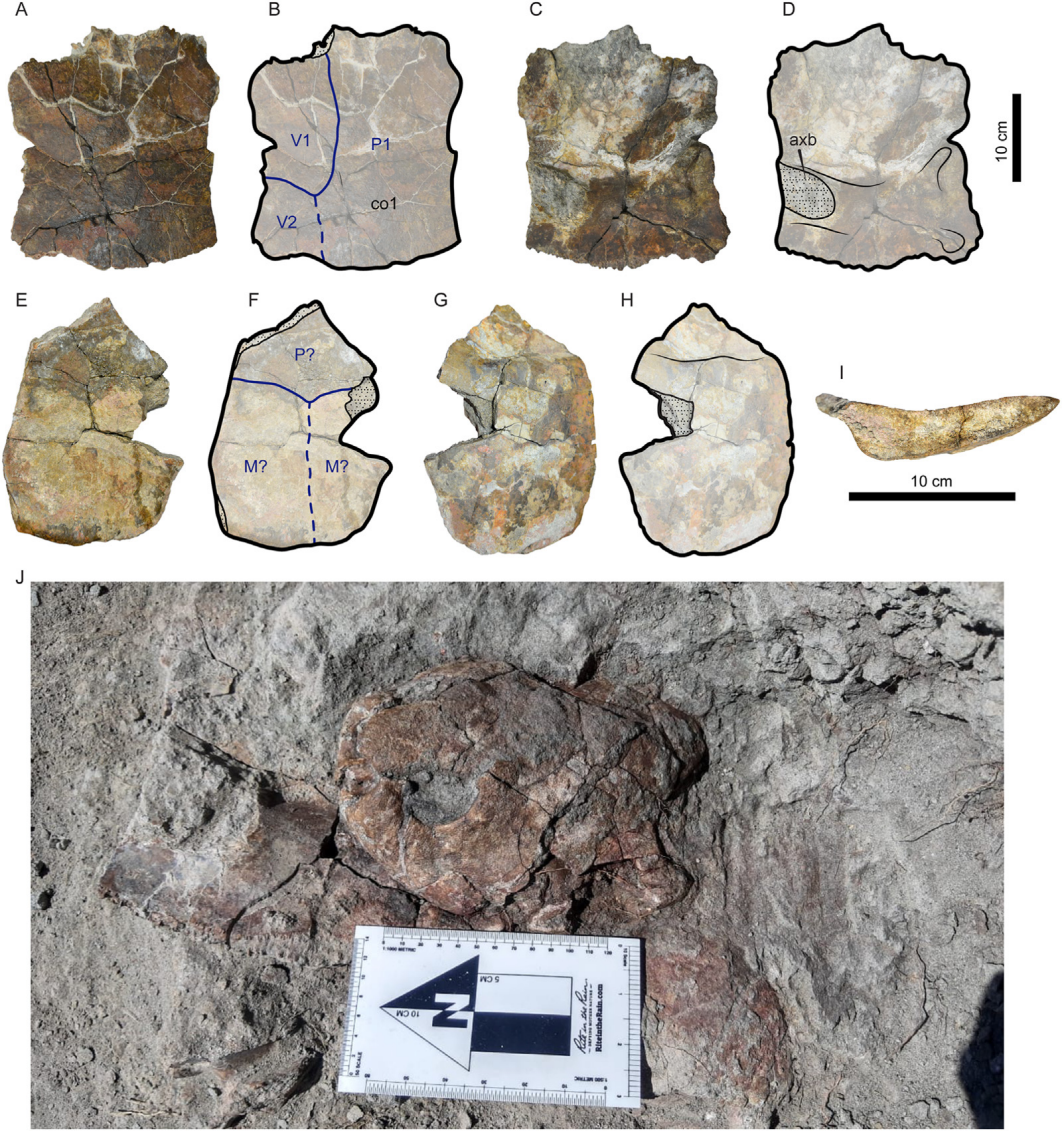
*Caninemys tridentata* VPPLT-1720 specimen, carapace fragments. (A–B) Costal 1 in dorsal view. (C–D) Costal 1 in ventral view. (E–F) Posterior peripheral in dorsal view. (G–H) Posterior peripheral in ventral view. (I) Posterior peripheral in lateral view. (J) *Caninemys tridentata* VPPLT-1720 specimen ‘in situ” discovered skull and carapace fragments associated. For abbreviations see Materials and Methods (numeral 2.5.).

*New referred material—*VPPLT-1720 represented by a nearly completely preserved skull (Figure 6), right costal 1, and posterior peripheral (Figure 7). Measurements of the skull, costal 1 and peripheral as preserved are in Table 1.

*Locality, stratigraphy and age*—VPPLT-1720 comes from the Repartidora locality (3º22′14.736″N, -75º8′46.032″W), lowermost segment of La Victoria Formation (Guerrero, 1997), dated as middle Miocene (13.6 +/− 0.2 Ma) (Cadena et al., 2020b) (Figure 5).

*Remarks***—**VPPLT-1720 belongs to *Caninemys tridentata* because it shares with the holotype DNPM-MCT-1496 (Meylan et al., 2009) the following features: 1) large and deep cavum pterygoidei; 2) greatly inflated maxillae; and 3) maxilla with a ventrolateral toothlike process, which, together with a single process formed on the midline of the premaxillae, form a tridentate condition in the upper triturating surfaces, unique among pleurodires (Meylan et al., 2009). An additional feature that is better preserved in VPPLT-1720 than the holotype is the temporal emargination margin, which tapers medially to form straight line margins similar to extant *Podocnemis* spp. (and differs from the almost fully roofed skull of *Peltocephalus dumerilianus* and *Erymnochelys madagascariensis*).

## 4 Descriptions

### 4.1 *Stupendemys geographica* specimen VPPLT-1337

*Skull*. The skull of VPPLT-1337 is broken at the level of the processus trochlearis pterygoidei  missing completely its midposterior region. The preserved portion of the skull has not suffered of crushing, only missing some small portions of the lateral margin of the right frontal and the most ventral margins of both maxillae. In dorsal view (Figure 1A–B), the skull exhibits converging lateral margins ending anteriomedially in a narrow straight edge, striated surface of skull roof bones particularly of prefrontals, frontals and parietals, and relatively small orbit cavities facing laterally. Prefrontals are large and forming the most anterior region of the snout, meeting each other medially, posteriorly in contact with frontals and posterolaterally contributing to form the orbit margin. Frontals meet each other medially and anteriolaterally contribute to the orbit margin, posteriolaterally in contact with parietals and postorbitals. Parietals meet each other medially, anterolaterally they contact the postorbitals, and ventrally descend to contact the pterygoids. Both postorbitals are also preserved being large and anteriorly contributing to the orbit margin, laterally in contact with jugals, and posterolaterally with quadratojugals. The most anterolateral portions of both quadratojugals are preserved being in contact with jugals anterolaterally. Well- preserved sulci indicate that the interparietal scute covered the posteromedial portion of frontals and projected tapering laterally onto parietals. The parietal scutes covered the lateral portions of parietals, posterior region of postorbitals, the posteromedial corner of jugals and most of quadratojugals.

In ventral view (Figure 1C–D), the skull of VPPLT-1337 shows a very deep concavity at the central portion of premaxillae. The upper triturating surface is formed by a medial contact between palatines and the posteromedial portion of premaxillae with a slight step at the posterolateral corners of the triturating surface. Premaxillae form the most anteromedial margin of the skull in this view, they meet each other medially, being very narrow right before the deep concavity, laterally contact the maxillae and posteriorly the palatines. Maxillae form the lateral half of the triturating surface exhibiting a pitted with small ridges bone surface. Palatines contact each other medially, laterally contact the maxillae at the upper triturating surface, and posteriorly they contact the pterygoids. The foramen palatinum posterius is large and located between the palatinopterygoid suture. The pterygoids contact each other medially, the palatines anterolaterally, and laterally form a well defined bar that ends with the processus trochlearis pterygoidei.

The anterior view of VPPLT-1337 (Figure 1E–F) shows the extreme dorsally inflated snout formed by the prefrontals, as well as the vertical wall that the prefrontals form above the narium externa aperture which is particually reduced. Both premaxillae meet each other anteromedially forming the floor of the narium externa and the lower part of the snout, posteriorly they contact the maxillae. In lateral view (Figure 1G–H), the contact between prefrontals and maxillae is very long. The orbital ring is formed by the contribution of the prefrontal, frontal, postorbital, jugal and maxilla. Jugals are in anterior contact with the maxillae, dorsally with the postorbitals and posteriorly with the quadratojugals. The sulci of the postorbital and parietal scutes are clearly defined.

*Carapace*. The carapace of VPPLT-1337 is slightly longer than wide (Figure 2, Table 1) missing most of its left anterolateral portion including parts of nuchal, costals and peripherals. It has a moderately height dome-shape in both anterior and posterior sides (Figure 3A–B). The anterior margin exhibits a relatively deep median notch and the contours of peripherals are irregular nodular in shape along the entire margin of the carapace. The dorsal bone surface of the carapace is smooth to slightly striate particularly at the level of right costals 2 and 3, which exhibit lines similar to growth annuli.

The most right portion of nuchal is preserved exhibiting a thickened and moderately upturned anterior margin (Figures 2A–B, 3C–E). The neural series is composed of six being neural 1 rectangular in shape and longer than wide, neurals 2 to 5 are hexagonals and neural 6 heptagonal in contact with costals 5 to 7. The costal series has eight pairs, being costal 1 the largest, and costals 7 and 8 meeting each other medially. There are eleven pairs of peripherals, being peripherals 1 and 2 extremely expanded anteriorly (Figures 3C–D), with peripheral 2 having a short protruding anteromedial margin. There is one suprapygal restricted between costals 8, peripherals 11 and the pygal. In general the sulci of the scutes are well preserved and deeply marked in the bones, there were five vertebral scutes being vertebral 1 the smallest of them and restricted anteriorly to the nuchal. Vertebrals 2 and 3 were octogonal slightly wider than long, vertebral 4 was heptagonal and vertebral 5 trapezoidal being slightly longer than wide. All the four pairs of pleural scutes reached the most anterior portions of the peripherals. There were twelve pairs of marginal scutes all them restricted to the peripherals, marginal 1 was large covering most of the nuchal and peripheral 1, and marginal 2 was particularly narrow at its posterior contact with pleural 1.

*Plastron*. The plastron of VPPLT-1337 is nearly complete and articulated to the carapace, only missing the most lateral margins of the left epiplastron, hyoplastron, mesoplastron and hypoplastron, as well as a small portion medial portion of xiphiplastra (Figure 2C–D). It is much shorter than the carapace without reaching any of its anterior and posterior margins (Table 1), and with both plastral lobes nearly of the same length. The right epiplastron is almost rectangular in shape showing a protruding lip at the extragular region. The entoplastron is diamond in shape with a convex posterior sutural contact with hyoplastra, reaching the level of the hyoplastra notch. The hyoplastra are slightly larger than hypoplastral with a longer medial contact between each other in contrast to medial hypoplastral contact. Mesoplastra are laterally restricted and having a nearly hexagonal shape. The anal notch is short in V-opened shape, suggesting that it might be represented a female.

The sulci of the plastral scutes are well defined and deeply marked on the bone. The right extragular was a small triangular scute restricted to the epiplastron. The intergular was relatively wide and reaching the most anterior corned of the entoplastron. Humerals had a short medial contact and were restricted to the epiplastra. Abdominal scutes were slighty longer than pectorals and femorals, only touching laterally the most anterior portion of mesoplastra. The anal scutes were restricted to the xiphiplastra, and there is not evidence of existence of inframarginals.

*Pectoral girdle*. The most distal regions of the right coracoid and the acromial process of scapula are preserved very close to their original anatomical position inside the shell and with their proximal portions covered by the anterior plastral lobe and embedded in the rock matrix (Figure 3F–G). The coracoid exhibits nearly the same width distally that at its median portion, only slightly flatter at its most distal end, it has a marked lateral ridge. The acromial process is tubular in shape keeping the same width along its length.

*Hindlimb*. Most of the right hindlimb bones are preserved disarticulated but close to their original anatomical position between the right plastral lobe and the carapace (Figure 3H–I). The femur is preserved in ventral view showing a relatively wide and flat tibial articulation area with a marked medial depression. The middle shaft region is slightly oval in its outline and the trochanter minor forms a thumblike rounded proximolateral projection. The concavity between trochanters is shallow. The tibia and fibula are missing. The astragalus, calcaneum and distal tarsal 4 are preserved articulated. The astragalus exhibits a curved surface for the articulation with the tibia. The calcaneum is a very small bone having slightly elongated shaped. Other preserved elements of the right pes are five relatively long metatarsals, four short phalanges and three digits.

*Cervical vertebrae*. Two cervical vertebrae are preserved for VPPLT-1337. The first corresponds to cervical 8, which is prevised very close to the right pectoral girdle bones (Figure 3F–G). Its centrum is short and the posterior condyle is oval in shape, the neural arch is high and portions of the postzygapophyses are very close between each other and with facets facing ventrally. The left lateral process of the vertebra is short and slightly facing ventrally. The ventral body of the vertebra forms a short and narrow flange.

The second preserved cervical vertebra is an isolated cervical 2 (Figure 3J–N). It is a opisthocoelous vertebra, with an anterior articulation being oval in shape slightly wider than tall, the transverse processes are long and ventrally projected, exhibits a tall neural arch and lacks of ventral keel (Figure 3J–K). The posterior articulation surface is of horse-saddle shaped, and showing postzypapophyses slightly separate from each other and with facets facing ventrally (Figure 3L–M). Dorsally the axis neural arch forms at its roof a flat to slightly convex surface, which becomes wider posteriorly (Figure 3N).

### 4.2 *Stupendemys geographica* specimen VPPLT-1719

*Carapace*. VPPLT-1719 is a partial carapace, preserving the nuchal, neurals 1 to 3, right costals 1 and 2, right peripherals 1 to 4, and portions of the left costals 1, 2, right costal 2, and right peripheral 5, the bone surface is mostly smooth with some striations at the sutural contact regions of bones (Figure 4A–D). It represents an adult specimen (Table 1). The anterior border of nuchal-peripheral 1 are thickened and strongly upturned (Figure 4E–F). The right peripherals 1 and 2 are extremely expanded anteriorly with peripheral 2 having a short protruding anteromedial margin. The cervical scute was absent, marginal 1 very large covering half of nuchal, peripheral 1 and reaching a portion of peripheral 2. Vertebral scute 1 was pentagonal and vertebral 2 octagonal. The pleurals 1 and 2 expanded onto the peripherals. In ventral view (Figure 4C–D), the axillary buttress scar is well developed onto the middle portion of costal 1 and projected laterally to reach peripheral 3, and the visceral surface where soft tissue was attached is marked by a groove that runs along the peripherals and nuchal. Thoracic vertebra 1 is also preserved, having a nearly circular and concave articular surface, and strong thoracic processes that reach costal 1 (Figure 4G–F, Supplementary File S5).

### 4.3 *Caninenys tridentata* specimen VPPLT-1720

*Skull*. It is nearly complete, missing most of its right otic chamber, and lateral portions of the left quadratojugal, jugal, and quadrate bones (Figure 6). The anterior roof of the skull formed by prefrontals and frontals is a slightly collapsed interrupting the orbital ring. The sutures are well defined and some of the sulci of the scutes that covered the skull are also identifiable. In dorsal view (Figure 6A–B), prefrontals meet medially and form most of the anterior roof of the skull, having a medially tapering anterior margin, they are part of the orbital rings and are in contact with maxillae ventrally and frontals posteriorly. Frontals also meet medially and anterolaterally form part of the orbital rings, posteriorly are in contact with parietals, and with postorbitals posterolaterally. Postorbitals are large and posteriorly in contact with quadratojugals and parietals, avoiding a contact between jugal and parietal. Parietals form most of the medial and posterior roof of the skull, meeting medially and having a long lateral contact with quadratojugal. The temporal emargination is formed by the posterior portion of parietals and covers most of the dorsal region of otic chambers, without a fully roofing of the posterior portion o the skull, its margins are straight, tapering medially to reach a small portion of the supraoccipital that is visible dorsally. The contact between the left quadrate, opistothic and squamosal is also visible in dorsal view. The sulci left by the interparietal scute indicate that was triangular in shape, posteriorly ending in acute tip, covering most of the medial region of the skull and reaching the most posterior portions of frontals. The frontal scute covered most of the frontals to reach the orbits.

In ventral view (Figure 6C–D), the skull of VPPLT-1720 shows a triangular in shape triturating surface, being posteriorly wider and formed mostly by the maxilla. There is one accessory ridge in each side of the surfaces. The vomer is preserved and sutured anteriorly to premaxillae, posteriorly ending in acute tip. Palatines meet each other medially and form most of the relatively flat palate. The foramen palatinum posterious is restricted to palatines, very close to the palatino-pterygoid suture. Both pterygoids are large, meeting medially and laterally exhibiting the processus trochlearis pterygoidei projected almost horizontally. The basisphenoid exhibits a triangular shape with some shallow depression on its ventral surface. The cavum pterygoidei or fossa pterygoidea is large and deep and better preserved in the left side of the skull formed by the pterygoid, opisthotic and quadrate. Also on the left side of the skull, the articular condyle is well preserved being longer than wide, and having a medial notch. The paroccipital process of the opisthotic is well defined and projected posteriorly beyond the squamosal. The basioccipital is well preserved and contributes together with the exoccipital to form the occipital condyle, anteriorly contacts the pterygoid via a semi-lunate depression.

The anterior view of VPPLT-1720 (Figure 6E–F), shows distinct premaxillary-maxillae toothlike processes projected ventrally in both sides of the skull, the tridentate-shape. The base of the fossa nasalis is formed by the premaxillae and the two praepalatinum formamina are enlarged and well preserved. The prefrontals form the roof of the large fossa nasalis, developing a hanging medial process ventrally projected. On the lateral surface (Figure 6G–H) of VPPLT-1720, the most remarkable feature is the massive maxilla that forms most of the anterior snout. Also the jugal is large reaching the orbit. The bones that form both cheek regions are broken allowing the observation of the processus trochlearis pterygoidei, but leaving unknown how well developed the cheek emargination was. Part of the cavum tympani is preserved showing the incisura columella auris enclosed by the quadrate, and its posterior portion includes a very small antrum postoticum. The descending process of the quadrate is short.

*Carapace*. Two pieces of the carapace of VPPLT-1720 are preserved (Figure 7). The first corresponds to the right costal 1, missing most of its lateral portion (Figure 7A–D). In dorsal view (Figure 7A–B), the sulci between vertebral scutes 1 and 2 are preserved indicating the vertebral 1 was slightly wider than vertebral 2, the surface of the bone is smooth without any sculpturing pattern. In ventral view (Figure 7C–D), the axillary buttress scar is preserved showing that was well developed until the medial region of the bone and posteriorly positioned. The other carapace bone preserved for VPPLT-1720 corresponds to a nearly complete peripheral bone (Figure 7E–I), from the posterior margin of the carapace based on the shape that exhibits laterally (Figure 7I), as in the costal bone, the bone surface is smooth, and the sulci between marginals and the pleural are preserved in dorsal view. These two bones were found right under the skull of VPPLT-1720 (Figure 7J), supporting that represent the first shell elements for *Caninemys tridentata*.

## 5 Discussion

### 5.1 A unique skull architecture among turtles

**Figure 8:**
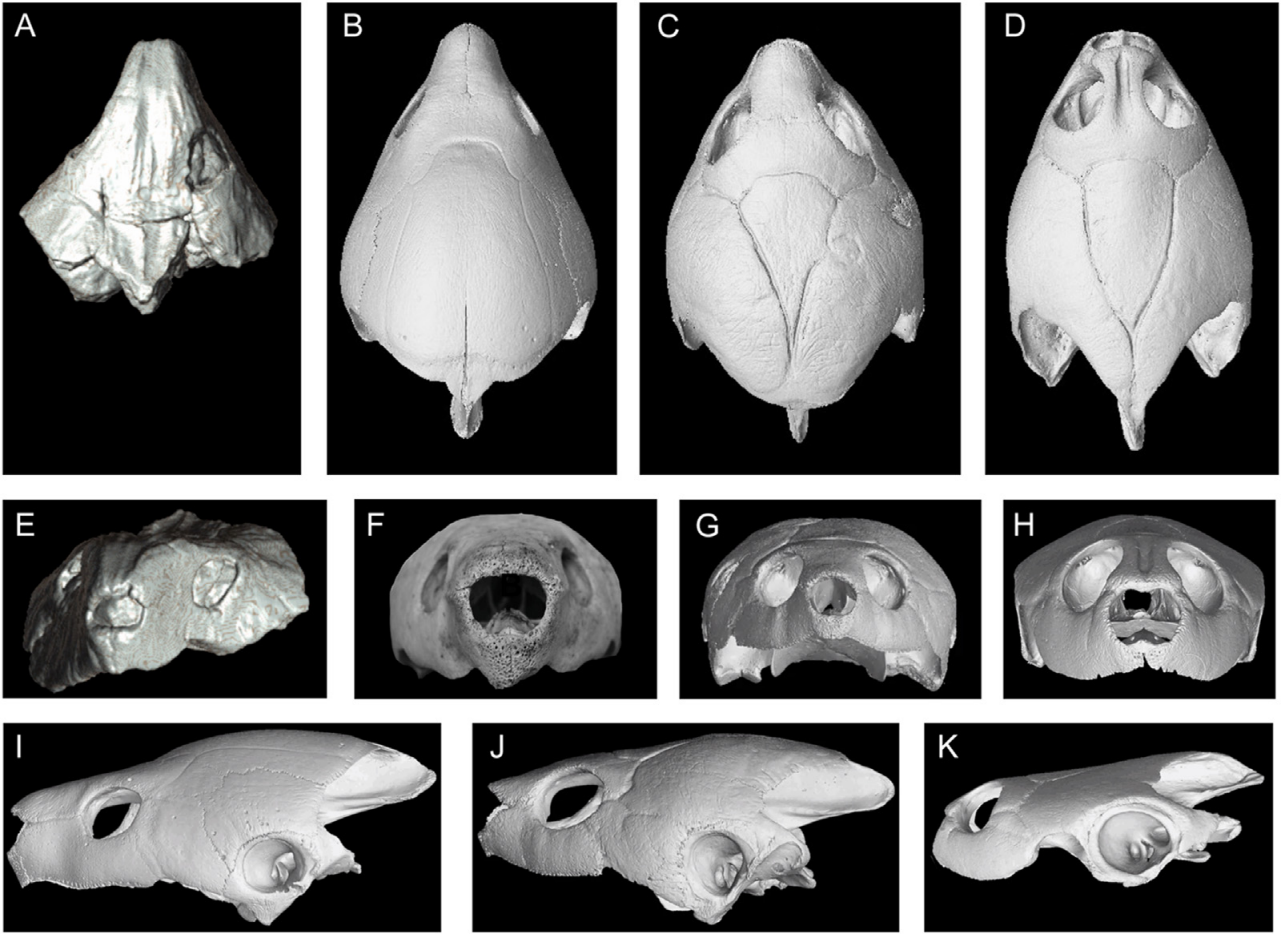
Skull comparisons among podocnemidids including *Stupendemys geographica*. (A–D) Skulls in dorsal view. (A) *Stupendemys geographica* VPPLT-1337, (B) *Peltocephalus dumerilianus* SMF-40169*,* (C) *Erymnochelys madagascariensis* SMF-33056*,* and (D) *Podocnemis unifilis* SMF-32915. (E–H) Skulls in anterior view. (E) *Stupendemys geographica* VPPLT-1337, (F) *Peltocephalus dumerilianus* SMF-40169*,* (G) *Erymnochelys madagascariensis* SMF-33056*,* and (H) *Podocnemis unifilis* SMF-32915. (I–K) Skulls in left lateral view. (I) *Peltocephalus dumerilianus* SMF-40169*,* (J) *Erymnochelys madagascariensis* SMF-33056*,* and (K) *Podocnemis unifilis* SMF-32915.

The architecture of the snout in the *Stupendemys geographica* VPPLT-1337 specimen is similar in many aspects to the snout of the extant *Peltocephalus dumerilianus*, particularly in having orbits facing laterally in dorsal view, an interparietal scute tapering posteriorly, long anteriorly projected prefrontals that fully cover the narium externa aperture, and a long contact between prefrontal and maxilla (Figure 8A–B, I). However, it differs from *Pe. dumerilianus*, *Erymnochelys madagascariensis*, and *Podocnemis unifilis* in having an extreme dorsal inflation of prefrontals, a descending vertical wall of prefrontals that cover the narium externa aperture, and a striated surface of the skull roof bones (Figure 8A–H). *Stupendemys geographica* VPPLT-1337 exhibits a very reduced narium externa aperture (Figure 8E), similar to some specimens of the extant *Pe. dumerilianus* (Figure 8F,I). The aperture is slightly larger in *E. madagascariensis* (Figure 8G,J) and much larger in *P. unifilis* (Figure 8H,K).

Another remarkable anatomical aspect of the skull of *S. geographica* comes from its anterior palate. In this region, *S. geographica* shares with *Pe. dumerilianus* a very deep concavity at the median region of the premaxillae (cranial pit). However, it differs from this taxon and all other podocnemidids in having an upper triturating surface formed by a medial contact between maxillae and palatines that creates a secondary palate, completely hiding the aperture narum interna (Figure 1C–D). A similar medial upper triturating surface is exhibited by *Dacquemys paleomorpha* Gaffney et al. (2002). However, in *D. paleomorpha* the medial region of the secondary palate is formed by the medial contact between maxillae, without contribution of the palatines as in *S. geographica*. Also, this upper triturating surface configuration is different from the secondary palate of other podocnemidid turtles, particularly of those included in the Stereogenyini clade (Ferreira et al., 2018), which exhibit a well-defined cavity that runs posteriorly from the medial region of the premaxillae, without developing a medial contact between maxillae and palatines on this surface.

**Figure 9:**
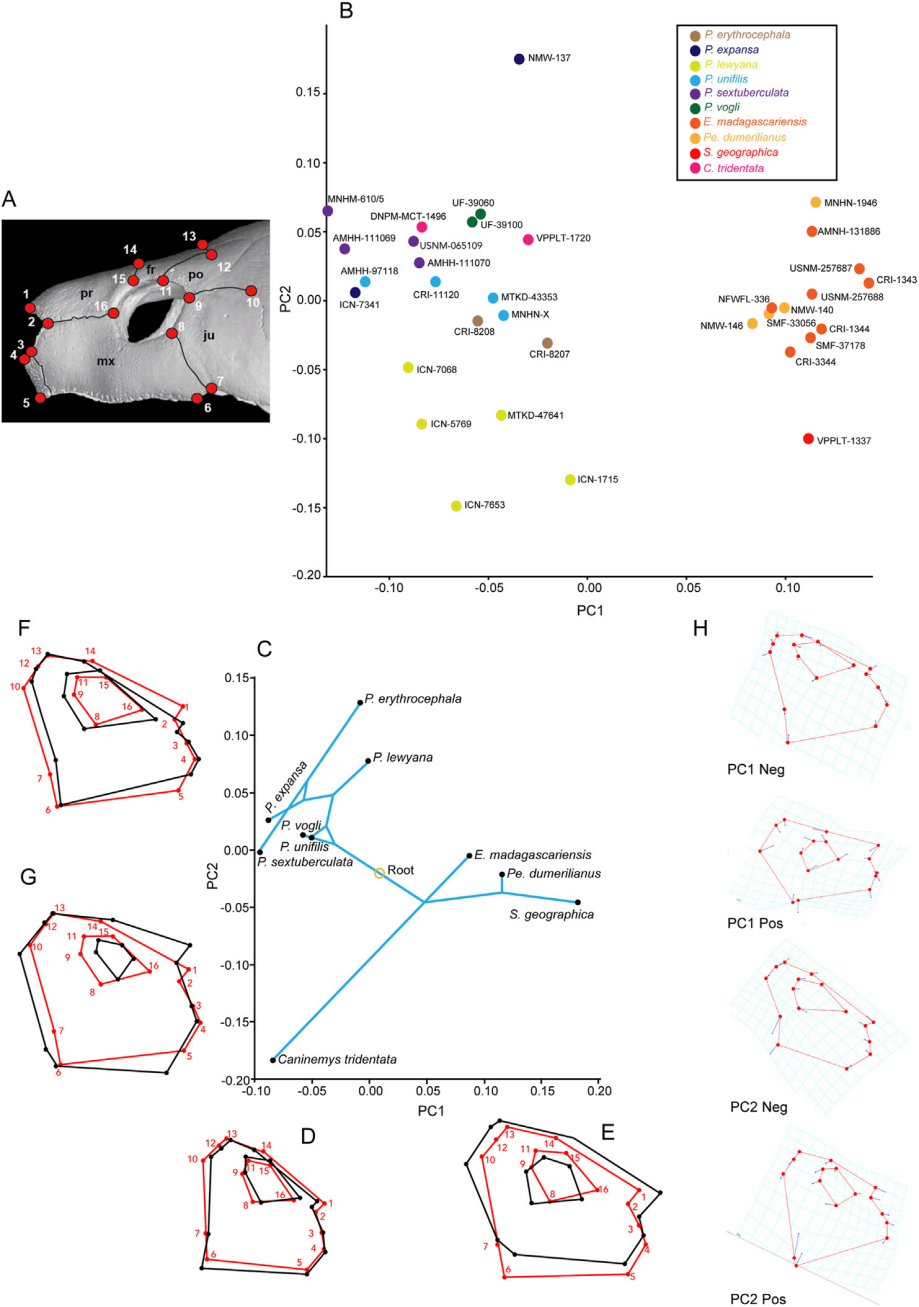
Geometric morphometric analysis of the lateral component of the snout in extant and some fossil podocnemidids. (A) Location of the 16 landmarks in the lateral view of the skull used in the analysis. (B) Plot of the first two principal components of a PCA of the extant and fossil taxa included, indicating the specimens examined (see list of specimens and institutional abbreviations in Supplementary File S1). (C) Phylomorphospace plot based on the strict consensus obtained (Supplementary File S6). (D) Variance of shape of the snout for the negative values of PC1, expressed by *Caninemys tridentata* and *Podocnemis* spp. (E) Variance of shape of the snout for the positive values of PC1, expressed by *Stupendemys geographica*, *Peltocephalus dumerilianus*, and *Erymnochelys madagascariensis*. (F) Variance of shape of the snout for the positive values of PC2, expressed by *Podocnemis* spp. (G) Variance of shape of the snout for the negative values of PC2, expressed by *Stupendemys geographica*, *Peltocephalus dumerilianus*, *Erymnochelys madagascariensis*, and the extreme shape of *Caninemys tridentata*. (H) The area of the morphospace occupied by *S. geographica.*

There are three major distinct snout architectures among the podocnemidid turtles studied, as supported by the geometric morphometric analysis that we conducted (Figure 9A). Figure 9B shows the first two principal components of a PCA of the extant and fossil taxa included. PC 1 accounts for 39.68% of the variation in the sample and divides the taxa from the extant *Podocnemis* spp plus *Caninemys tridentata* at the negative end of the axis from *Erymnochelys*, *Peltocephalus* and *Stupendemys* (VPPLT-1337 specimen) placed at the positive end. PCA 1 is driven largely by the position of the orbit, the anterior projection of the snout, the overall height of the skull, and the position of landmark 7 (jugal-maxilla at the cheek emargination). PC 2 contains 17.12% of the variation in the sample and largely separates one specimen of *P. expansa* at the positive end, and all *P. lewyana* at the negative end of the axis. PC 2 is driven by the shape of the orbit, the slope of the dorsal most aspect of the skull, and particularly the position of landmark 1 (anteriormost tip of premaxillae) and landmark 7 (jugal-maxilla at the cheek emargination). *S. geographica* occupies a unique portion of the morphospace, grouping with the *Peltocephalus* and *Erymnochelys* on PC 1 and *P. lewyana* on the negative end of PC 2.

There is a significant (p = 0.0215) component of shape related to phylogeny. In the phylomorphospace plot (Figure 9C), 40.6% of the variation is contained on PC 1 and 29.1% is contained on PC 2. PC 1 is related to the relative size of the orbit as compared to the height of the anterodorsal portion of the skull (prefrontals-frontal). *C. tridentata* and *Podocnemis* spp. fit on the negative end of the axis having relatively small orbits and very narrow prefrontal-frontal lateral exposure (Figure 9D); whereas all of the other taxa have the opposite morphology (Figure 9E). PC 2 separates all of the species in the genus *Podocnemis* from the rest of the sample and is related to larger size of the orbit and the shape of the anterodorsal roof of the skull. *Podocnemis* species have larger orbits and a very narrow width between orbit and cheek emargination (Figure 9F), whereas individuals on the negative part of the axis, such as *Erymnochelys*, *Peltocephalus*, *Stupendemys,* and *Caninemys* are typified by a relatively smaller orbit and wider bone surface between the orbit and the cheek emargination level (Figure 9G). Shape changes for each PC1 and PC2 negative and positive are shown in Figure 9H.

### 5.2 The ontogeny of *Stupendemys geographica*

The VPPLT-1337 specimen of *Stupendemys geographica* represents the first fossil shell measuring less than 1 m long. All previously described shells have measured greater than 1 m and reach a maximum of 2.86 m (Cadena et al., 2020a). This new and smallest known specimen (at the time of publication) of *S. geographica* offers the opportunity to establish some insights into the ontogenetic changes of the shell and postcranial elements that possibly took place in this extinct giant turtle. We designate this specimen as a juvenile to early adult female based on its size, lack of massive horns (Cadena et al., 2020a), and shallow anal notch in the plastron.

Features that remain without major changes from juvenile-adult to fully adult stages in *S. geographica* include: the occurrence of a median nuchal notch; the anterior expansion of peripherals 1 and 2; the irregular nodular contours of the carapace; medial contact between costals 7 and 8; relative size between plastral lobes; plastral scute arrangement except the pectorals; and the general morphology of the cervical 8 vertebra, particularly the articular facets of postzygapophyses forming an angle of less than 90°.

**Figure 10:**
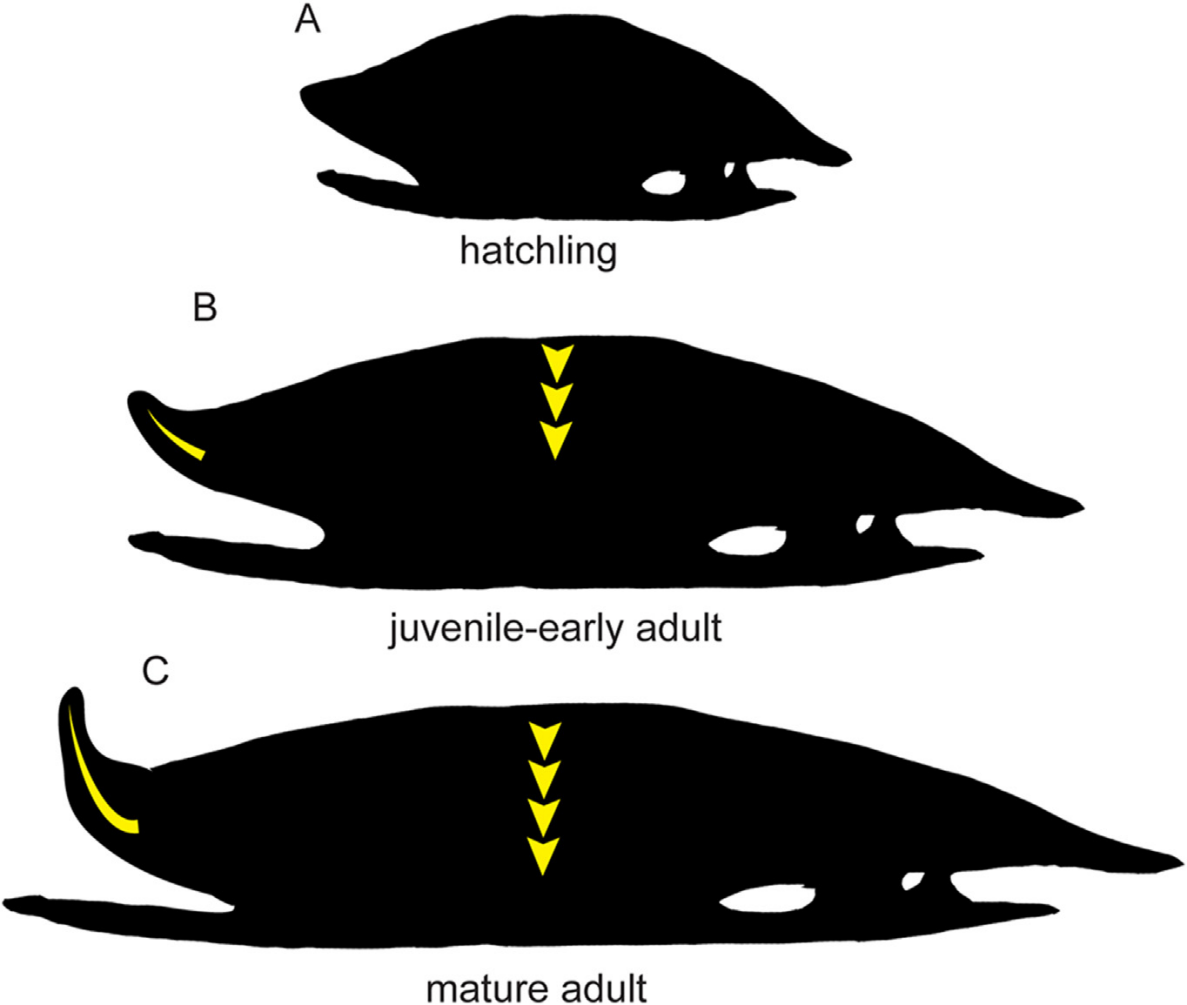
Representation of the possible ontogenetic changes in the shell of a female individual of *Stupendemys geographica.* (A) hypothetical hatchling. (B) juvenile to early adult based on VPPLT-1337. (C) mature adult based on VPPLT-1719 and specimens described in Cadena et al. (2020a).

Comparisons between *S. geographica* VPPLT-1337 and adult specimens described in Wood (1976) and Cadena et al. (2020a) show some significant ontogenetic changes in this taxon. The first remarkable change is in the height of the carapace, as it is flatter in adults (Figure 10). This follows a similar pattern to the extant freshwater turtle *Trachemys scripta elegans* (Fish and Stayton, 2014). The architecture of the nuchal region of the carapace also changes ontogenetically, with a more pronounced upturned anterior margin of the nuchal and peripheral 1 in the adult specimens (Figure 10), creating a wider and deeper anteromedial carapace embayment.

**Figure 11:**
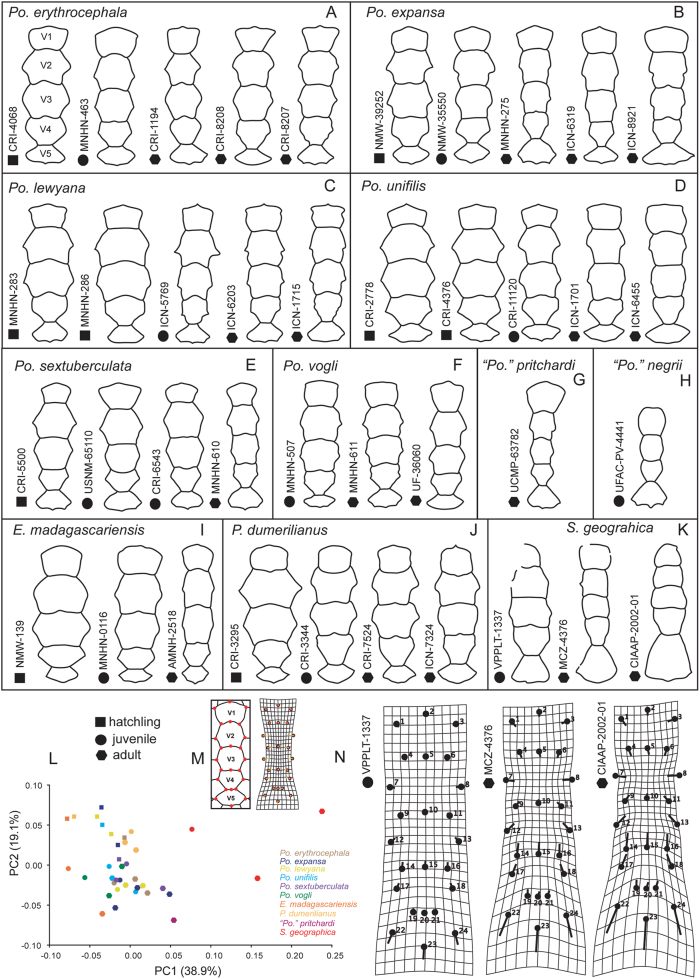
Variation of the vertebral scutes in all species studied at all ontogenetic levels. (A–K) ontogenetic variation of the five vertebral scutes of the carapace in hatchling (squares), juveniles (circles) and adults (hexagons). (A) *Podocnemis erythrocephala*, (B) *P. expansa*, (C) *P. lewyana,* (D) *P. unifilis,* (E) *P. sextuberculata,* (F) *P. vogli,* (G) “*P”. pritchardi,* (H) “*P”. negrii,* (I) *Erymnochelys madagascariensis,* (J) *Peltocephalus dumerilianus,* (K) *Stupendemys geographica*. (L) Principal component analysis from the geometric morphometric analysis showing the large difference between *S*. *geographica* and the other taxa included in terms of vertebral scutes shape. (M) Location of the 24 landmarks in the vertebral scutes of the carapace used in the analysis. (N) Grid model for the five vertebral scutes in three different specimens of *Stupendemys geographica*, showing the transformation that each individual has from the average *Podocnemididae* shape for the 25 landmarks. For abbreviations see Materials and Methods (numeral 2.5.).

In terms of the morphological variability of the vertebral scutes in our sample, vertebrals 2 and 3 decrease in size during development, becoming narrower from hatchling to adult stages in almost all specimens of *Podocnemis* spp. (Figure 11A–F, L–M), *Erymnochelys madagascariensis* (Figure 11I, L–M), *Peltocephalus dumerilianus* (Figure 11J, L–M), and also between the VPPLT-1337 juvenile-adult to adult specimens of *S. geographica* (Figure 11K, L,N). The sole specimens of “*Podocnemis” pritchardi* (Wood, 1997) (Figure 11G, L) and “*Podocnemis*” *negrii* Carvahlo et al. (2002) (Figure 11H), which possibly represent adults of each taxon, also exhibit narrower vertebrals 2 and 3. Another aspect of the vertebrals that changes with ontogeny in *S. geographica* is the length of vertebral 5, which becomes the longest and widest of the series in adults, keeping its trapezoidal distinct shape (Figure 11K, L,N). The attribution of “*Podocnemis” pritchardi* and “*P*.” *negrii* to the extant genus *Podocnemis* by Gaffney et al. (2011) has been questioned because this genus lacks indisputable diagnostic shell characters and can only be distinguished from other podocnemidids by skull characters. The principal component analysis performed on the geometric morphometric analysis of vertebrals (Figure 11L) indicates that “*P.*” *pritchardi* is an adult and exhibits a distinct vertebral scute pattern in contrast to all other podocnemidids included in the analysis, particularly by an enlarged vertebral 1 as compared to the rest of the sequence and very narrow vertebrals 2 to 4. A feature that not only lacks significant ontogenetic change in *S. geographica* but also exhibits a similar shape and pattern to *Pe. dumerilianus* is vertebral 1. In both taxa, this scute has a nearly square shape with parallel to slightly anteriorly convergent margins (Figure 11J–K), in contrast to *Podocnemis* spp., *Erymnochelys madagascariensis* and “*P*.” *pritchardi*, which exhibit a vertebral 1 nearly pentagonal in shape with lateral margins anteriorly diverging (Figure 11A–K, I).

Based on these results, characters previously used by phylogenetic analyses related to the width between vertebrals versus pleural scutes (i.e., Cadena, 2015: character 154; Hermanson et al., 2020: characters 203, 205, 208, 209, 210) should be carefully considered because they exhibit high ontogenetic variability within podocnemidids. Depending on the taxa involved in the analyses, the polymorphism should be coded or even excluded from analyses.

### 5.3 The skull of *Caninemys tridentata*

The discovery of the *Stupendemys geographica* VPPLT-1337 specimen, which constitutes the first individual of this taxon for which skull and shell have been found associated, allows us to reevaluate a recent attribution of the skull-based taxon *Caninemys tridentata* described by Meylan et al. (2009) to *S. geographica* (Cadena et al., 2020a). This hypothesis was based on the potential complementary anatomy between the lower jaws from Urumaco (Venezuela), La Venta (Colombia), and Acre (Brazil) and the skull of *C. tridentata*, particularly of the upper and lower triturating surfaces (Cadena et al., 2020a). This was additionally supported from original suggestions made by Meylan et al. (2009). We were able to test this hypothesis with the discovery of the VPPLT-1337 specimen and demonstrate that it is in part erroneous as the snout and upper triturating surface architecture of the *C. tridentata* and *S. geographica* (VPPLT-1337) skulls vary considerably (Figure 9). Differences that are unlikely to be ontogenetic or sexually dimorphic in nature.

### 5.4 Phylogenetic position of *Stupendemys geographica*

The first known skull and shell from the same individual of *Stupendemys geographica* offered us the opportunity to revise its phylogenetic position inside Pan-Pleurodira. The first analysis (all taxa, all characters; see methods) produced 27 most parsimonious trees (MPT), from which a strict consensus was obtained (Supplementary File S6). However, the support values for this analysis were poor: Tree Length (TL) = 1315; Consistency Index (CI) = 0.272; and Retention Index (RI) = 0.738. This resembled the topology presented in Joyce et al. (2021) with the exception of a major polytomy of the *Podocnemis* spp. and different relationships inside the clade formed by *Erymnochelys madagascariensis, Neochelys* spp*. Turkanemys pattersoni, Kenyemys williamsi* and *Papoulemys laurenti*. All of these differences are expected due to an update in coding we performed, which involved fixing some errors and coding for polymorphisms related to ontogeny as discussed above. In this strict consensus, *Caninemys tridentata* and *Stupendemys geographica* were found in different positions inside a larger clade that included the extant *Peltocephalus dumerilianus*, *Bairdemys* spp., and other taxa of the Stereogenyini clade following Ferreira et al. (2018). This supports the claim that these two specimens correspond to different taxa with two completely different skull morphologies.

**Figure 12:**
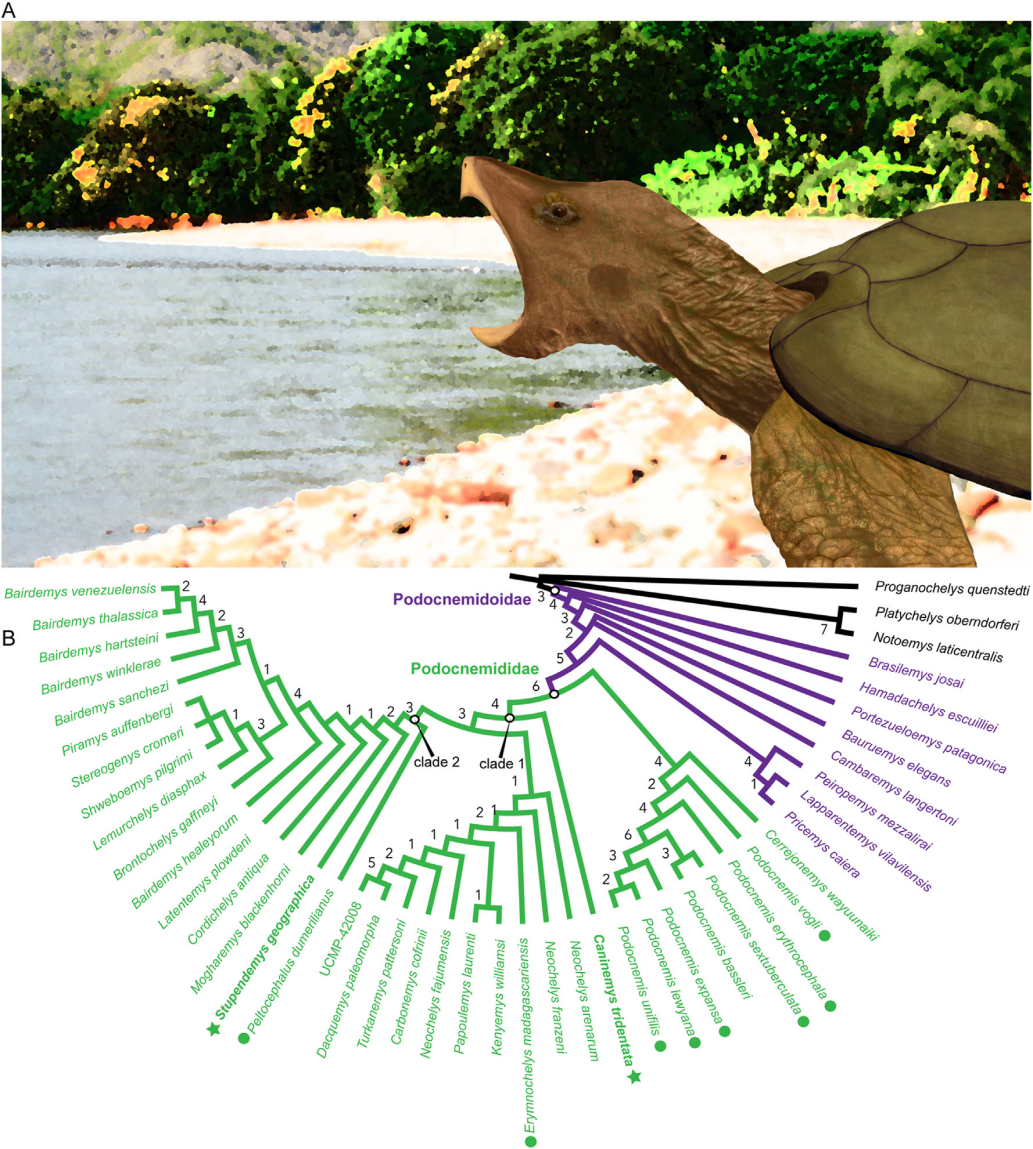
Restoration of a juvenile female individual of *Stupendemys geographica* and its phylogenetic relationships. (A) Restoration of *Stupendemys geographica* based on VPPLT-1337 specimen described herein, inhabiting a riverine environment of the Pebas system during the Miocene. (B) Our preferred phylogenetic hypothesis of the relationships among *Podocnemididae*, including *Stupendemys geographica* and *Caninemys tridentata.* Bremer support index showed at the base of the clades.

The second analysis excluded many taxa of Pan-Pleurodira and focused only on the Podocnemidoidae clade (taxa from *Brasilemys josai* and above). This analysis produced a single most parsimonious tree (Figure 12B): TL = 464; and improved values of CI = 0.772, and RI = 0.971. Thus, this is our preferred phylogenetic hypothesis and topology to explain the possible relationships of *C. tridentata* and *S. geographica* within the *Podocnemididae* clade. *C. tridentata* is at the base of a large clade (Clade 1, Figure 12B) that includes the extant *E. madagascariensis, Pe. dumerilianus*, and their relatives. This clade is separated from Podocnemidinae (*Podocnemis* spp. plus the fossil *Cerrejonemys wayuunaiki*) and is supported by more advanced cover of the adductor fossa (temporal emargination) (character 19); premaxillae reaching aperture narum interna (character 47); a large anterior opening of the cavum pterygoidei (character 96); and the lack of a horizontal occipital shelf (character 117). *S. geographica* is found inside a clade that also includes the extant *Pe*. *dumerilianus* at its base (Clade 2, Figure 12B), supported by: interparietal scutes without meeting medially (character 29); premaxillae having a cranial pit on the ventral surface (character 46); and a foramen palatinum posterius located at the sutural contact between palatine and pterygoid (character 71).

### 5.5 Two giant turtles in a single habitat

The competitive exclusion principle (Gause, 1934), also known as Gause's law (Pocheville, 2015) states that “complete competitors cannot coexist” or “ecological differentiation is the necessary condition for coexistence” (Hardin, 1960). Exploring competitive exclusion or opposite niche coexistence in fossil taxa is important because biotic interactions play a key role in structuring biodiversity patterns over large spatiotemporal scales (Fraser et al., 2021). In the fossil record of vertebrates, these kinds of ecological questions have been addressed in pterosaurs, bats, and birds (McGowan and Dyke, 2007), northern South American crocodylians (Scheyer et al., 2013), multituberculate mammals (Adams et al., 2019), and megaherbivorous dinosaurs (Mallon, 2019) among others. These new finds from the Colombian Tatacoa Desert demonstrate that two giant freshwater turtles (*Stupendemys geographica* and *Caninemys tridentata*) coexisted in the same habitat (Pebas wetland system) during part of the middle to late Miocene; despite being similar in size, with larger individuals reaching more than 2 m long. The completely different skull morphologies of *C. tridentata* and *S. geographica* indicate also potentially different paleodiets for each taxon. A vacuum feeding strategy combined with a powerfully biting supported by the three toothlike structures of the maxillae in *C. tridentata* (Meylan et al., 2009); and a more omnivorous-durophagous behavior for *S. geographica* based on the lower jaw (Cadena et al., 2020a) and skull described herein. Excluding dietary competition, these species might still have competed for nesting or basking space; however, this is unlikely in the extensive and interconnected Pebas system (Hoorn et al., 2010; Espinosa et al., 2021), another factor that potentially allowed the coexistence of both taxa.

### 5.6 Limitations of the study

Completeness of fossil specimens so far known. Only one skull-shell individual for *Stupendemys geographica* and two skulls, one with some carapace pieces, associated for *Caninemys tridentata*. Also, the missing portions of the skull of *S. geographica,* preventing the acquisition of key information from its posterior region. As such, whereas the conclusions given represent most likely interpretations, they are not definitive.

## Declarations

### Author contribution statement

Edwin-Alberto Cadena & Melissa Tallman: Conceived and designed the experiments; Performed the experiments; Analyzed and interpreted the data; Contributed reagents, materials, analysis tools or data; Wrote the paper.

Andrés Link, Siobhán B. Cooke, Laura K. Stroik & Andrés Vanegas: Contributed reagents, materials, analysis tools or data; Wrote the paper.

### Funding statement

Dr Edwin-Alberto Cadena and Dr. Andrés Link were supported by Fundación Para La Promoción de la Investigación y la Tecnología, Banco de la República de Colombia (4381).

Dr. Edwin-Alberto Cadena was supported by 10.13039/501100008793Universidad del Rosario (Fondo Capital Semilla IV-FCS018, 2019).

Dr. Siobhán B. Cooke, Dr. Melisa Tallman, and Dr. Laura K. Stroik were supported by the 10.13039/100005966Leakey Foundation.

Dr. Siobhán B. Cooke was supported by National Geographic Foundation.

### Data availability statement

Data included in article/supplementary material/referenced in article.

### Declaration of interests statement

The authors declare no conflict of interest.

### Additional information

No additional information is available for this paper.

## Appendix A Supplementary data

Supplementary_FileS1.docx Supplementary_FileS1.docx

Supplementary_FileS2.docx Supplementary_FileS2.docx

Supplementary_FileS3.docx Supplementary_FileS3.docx

Supplementary_FileS4.nex Supplementary_FileS4.nex

Supplementary_FileS5.docx Supplementary_FileS5.docx
