# Supplementary material for: New insights on the anatomy and ontogeny of the largest extinct freshwater turtles
Source: Heliyon. 2021 Dec 27;7(12):e08591. doi: 10.1016/j.heliyon.2021.e08591 (PMC8717240; doi:10.1016/j.heliyon.2021.e08591)
Supplement: Supplementary_FileS1.docx [file mmc1.docx]

**Supplementary File S1 for**

**New insights on the anatomy and ontogeny of the largest extinct freshwater turtles**

Edwin-Alberto. Cadena^1,2,*^, Andrés Link^3^, Siobhán B. Cooke^4^, Laura K. Stroik^5^, Andrés F. Vanegas^6^, Melissa Tallman^5^

^1^Universidad del Rosario, Facultad de Ciencias Naturales, Grupo de Investigación Paleontología Neotropical Tradicional y Molecular (PaleoNeo), Bogotá, Colombia

^2^ Smithsonian Tropical Research Institute, Panamá, Republic of Panama

^3^ Departamento de Ciencias Biológicas, Universidad de Los Andes, Bogotá, Colombia

^4^ Center for Functional Anatomy and Evolution, Johns Hopkins University School of Medicine, Baltimore, MD, USA

^5^Department of Biomedical Sciences, Grand Valley State University, Allendale, MI, USA

^6^Museo de Historia Natural la Tatacoa, La Victoria, Huila, Colombia.

**^*^Correspondence:** e-mail: edwin.cadena@urosario.edu.co (E-A. C)

**File S1. List of extant and fossil specimens examined**

List of specimens directed examined by the senior author.

**Institutional abbreviations**—AMNH, American Museum of Natural History, New York, USA; CRI, Chelonian Research Institute, Oviedo, Florida, USA; ICN, Instituto de Ciencias Naturales, Universidad Nacional de Colombia, Bogotá, Colombia; MNHN, Muséum National D’Histoire Naturelle , Paris, France; MTKD, Senckenberg Natural History Collections, Dresden, Germany; NFWFL, National Fish and Wildlife Foundation, Herpetological collection, University of Florida, Gainesville, FL, USA; NMW, Naturhistoriches Museum Wien, Viena, Austria; SMF, Senckenberg Natural History Collections, Frankfurt, Germany; USNM, Smithsonian National Museum of Natural History, herpetology collection, Washington, USA; UF, University of Florida Herpetological Collection, Gainesville, FL, USA; YM, Yale University Museum (Peabody), New Haven, CT, USA.

**Podocnemididae**

| Taxon | Collection number | Preservation |
| --- | --- | --- |
| Erymnochelys madagascariensis | AMNH-843/20 | Skull |
|  | AMNH-2518 | Shell |
|  | AMNH-63579 | Carapace, plastron, postcrania |
|  | MNHN-1897-80 | Skull, jaw |
|  | MNHN-2002-0116 | Shell |
|  | MNHN-zootheca | Shell |
|  | MNHN-1946-71 | Skull, jaw, shell |
|  | NMW-139 | Skull, jaw, shell |
|  | NMW-140 | Skull, jaw |
|  | NMW-141 | Skull, jaw |
|  | NMW-142 | Skull, jaw |
|  | NMW-146 | Skull, jaw |
|  | NMW-147 | Skull, jaw |
|  | NMW-422 | Postcrania |
|  | NMW-1811 | Complete skeleton |
|  | NMW-1812 | Complete skeleton |
|  | NMW-1813 | Complete skeleton |
|  | NMW-1814 | Complete skeleton |
|  | NMW-1815 | Complete skeleton |
|  | NMW-1816 | Complete skeleton |
|  | NMW-1821 | Complete skeleton |
|  | NMW-1822 | Complete skeleton |
|  | NMW-1839 | Complete skeleton |
|  | NMW-1840 | Complete skeleton |
|  | SMF-33056 | Skull CT scanned |
|  | YM-15398 | Skull, jaw, postcrania |
| Peltocephalus dumerilianus | AMNH-131886 | Skull, jaw, shell, postcrania |
|  | AMNH-785-PU-ost | Shell |
|  | CRI-7524 | Shell |
|  | CRI-3344 | Skull, jaw, shell |
|  | CRI-3295 | Skull, jaw, shell |
|  | CRI-1348 | Skull |
|  | CRI-1344 | Skull |
|  | CRI-1343 | Skull |
|  | ICN-7631 | Shell |
|  | ICN-7324 | Skull, jaw, shell |
|  | NFWFL-339 | Skull |
|  | NFWFL-337 | Skull |
|  | NFWFL-336 | Skull |
|  | SMF-37178 | Skull, CT scanned |
|  | USNM-257688 | Skull |
|  | USNM-257687 | Skull |
| Podocnemis erythrocephala | CRI-1194 | Shell |
|  | CRI-1922 | Shell |
|  | CRI-2757 | Shell |
|  | CRI-4068 | Shell |
|  | CRI-6023 | Shell |
|  | CRI-8207 | Skull, shell |
|  | CRI-8208 | Skull, shell |
|  | ICN-7339 | Shell |
|  | MNHN-1463 | Shell |
|  | MTKD-40660 | Skull, shell, postcrania |
|  | UF-57921 | Skull, postcrania |
|  | USNM | Skull, jaw |
| Podocnemis expansa | AMNH-58098 | Skull, jaw |
|  | AMNH-62947 | Shell, postcrania |
|  | AMNH-97124 | Skull, jaw |
|  | ICN-6319 | Shell |
|  | ICN-7341 | Skull, jaw, shell |
|  | ICN-7372 | Complete specimen |
|  | ICN-X | Skull |
|  | MNHN-275 | Skull, jaw, shell, postcrania |
|  | NMW-1824 | Complete skeleton |
|  | NMW-1852 | Complete skeleton |
|  | NMW-34528 | Complete skeleton |
|  | NMW-137 | Skull, jaw |
|  | NMW-35550 | Complete skeleton |
|  | UF-118589 | Skull |
|  | USNM-29476 | Shell |
|  | USNM-65112 | Postcrania |
|  | USNM-65113 | Skull |
| Podocnemis lewyana | CRI-3230  ICN-7653 | Shell  Complete skeleton |
|  | ICN-7068 | Skull, jaw |
|  | ICN-6468 | Carapace |
|  | ICN-6318 | Plastron |
|  | ICN-6207 | Complete skeleton |
|  | ICN-6203 | Shell |
|  | ICN-5769 | Skull, jaw, shell |
|  | ICN-1715 | Skull, jaw, shell, postcrania |
|  | ICN-1699 | Skull, jaw, shell, postcrania |
|  | ICN-1672 | Shell |
|  | MNHN-1944-286 | Shell |
|  | MNHN-1944-283 | Shell |
|  | MTKD-47641 | Skull |
| Podocnemis sextuberculata | MNHN 610/5 | Skull, jaw, shell |
|  | AMNH 111068 | Skull |
|  | AMNH 111069 | Skull |
|  | AMNH 111070 | Skull |
|  | USMN 065109 | Skull, jaw |
|  | USNM 065110 | Skull, jaw, shell |
|  | CRI 2830 | Shell |
|  | CRI 5500 | Skull, shell |
|  | CRI 6543 | Articulated shell |
| Podocnemis unifilis | AMNH-58195 | Skull |
|  | AMNH-97118 | Skull, jaw |
|  | AMNH-97118 | Skull, jaw |
|  | CRI-2776 | Shell |
|  | CRI-2778 | Shell |
|  | CRI-4376 | Shell |
|  | CRI-11120 | Skull, jaw, postcrania |
|  | ICN-1701 | Skull, shell |
|  | ICN-1721 | Shell |
|  | ICN-6455 | Skull, shell, postcrania |
|  | MNHN-X | Skull, jaw, shell |
|  | MTKD-42825 | Shell |
|  | MTKD-43353 | Skull, jaw, shell, postcrania |
|  | MTKD-45847 | Skull, jaw, shell, postcrania |
|  | NMW-1824 | Complete skeleton |
|  | SMF-37178 | Skull, CT scanned |
|  | USNM-313861 | Skull, shell |
| Podocnemis vogli | ICN-1682 | Complete specimen |
|  | MNHN-A507A | Shell |
|  | MNHN-X1 | Skull, jaw, shell |
|  | UF-39060 | Skull, jaw, shell |
|  | UF-39100 | Skull, jaw, shell |
|  | USNM-266206 | Skull, jaw |
