# Supplementary material for: New insights on the anatomy and ontogeny of the largest extinct freshwater turtles
Source: Heliyon. 2021 Dec 27;7(12):e08591. doi: 10.1016/j.heliyon.2021.e08591 (PMC8717240; doi:10.1016/j.heliyon.2021.e08591)
Supplement: Supplementary_FileS2.docx [file mmc2.docx]

**Supplementary File S2 for**

**New insights on the anatomy and ontogeny of the largest extinct freshwater turtles**

Edwin-Alberto. Cadena^1,2,*^, Andrés Link^3^, Siobhán B. Cooke^4^, Laura K. Stroik^5^, Andrés F. Vanegas^6^, Melissa Tallman^5^

^1^Universidad del Rosario, Facultad de Ciencias Naturales, Grupo de Investigación Paleontología Neotropical Tradicional y Molecular (PaleoNeo), Bogotá, Colombia

^2^ Smithsonian Tropical Research Institute, Panamá, Republic of Panama

^3^ Departamento de Ciencias Biológicas, Universidad de Los Andes, Bogotá, Colombia

^4^ Center for Functional Anatomy and Evolution, Johns Hopkins University School of Medicine, Baltimore, MD, USA

^5^Department of Biomedical Sciences, Grand Valley State University, Allendale, MI, USA

^6^Museo de Historia Natural la Tatacoa, La Victoria, Huila, Colombia.

**^*^Correspondence:** e-mail: edwin.cadena@urosario.edu.co (E-A. C)

**File S2. Landmarks of the vertebral scutes and skull**

Vertebral scutes

LM=24

100 104

165 88

234 102

118 170

161 165

206 170

73 236

245 232

102 290

152 278

213 290

74 346

241 350

105 408

156 404

208 405

85 450

230 453

128 508

154 508

178 506

88 540

156 573

225 556

ID=Erythrocephala_H

LM=24

332 121

402 93

481 122

356 197

402 186

444 201

326 266

469 265

350 317

396 308

445 316

330 374

466 374

356 425

398 425

442 424

341 466

458 466

378 514

402 513

426 510

338 546

408 581

472 546

ID=Erythrocephala _J

LM=24

588 104

638 89

697 102

609 186

638 188

665 184

576 240

701 234

602 297

637 288

674 292

577 352

706 342

608 416

646 405

681 412

594 448

704 441

629 502

653 505

674 504

596 549

662 574

725 541

ID=Erythrocephala _A1

LM=24

825 105

894 88

969 108

854 174

890 173

922 174

816 230

962 228

846 300

888 293

933 298

820 350

957 349

842 414

889 420

929 414

829 464

949 461

865 520

884 520

904 520

825 550

888 576

952 554

ID=Erythrocephala _A2

LM=24

1061 100

1122 86

1193 102

1084 176

1121 174

1157 177

1048 228

1192 232

1074 293

1117 292

1162 297

1046 348

1185 348

1069 406

1113 404

1156 408

1053 448

1169 448

1088 505

1109 506

1132 506

1046 549

1106 576

1172 550

ID=Erythrocephala _A3

LM=24

1378 113

1437 90

1499 110

1389 177

1441 169

1495 173

1358 238

1519 232

1391 290

1439 286

1491 280

1361 361

1518 350

1389 405

1441 406

1497 409

1377 465

1506 461

1418 509

1442 512

1467 509

1382 540

1443 574

1509 540

ID=Expansa_H

LM=24

1599 110

1659 88

1723 108

1622 174

1663 170

1702 173

1597 240

1741 242

1619 302

1665 294

1707 297

1602 353

1731 353

1623 413

1666 412

1711 409

1603 458

1722 456

1641 497

1661 500

1681 498

1598 538

1657 570

1721 533

ID=Expansa_J

LM=24

1817 114

1879 85

1945 108

1839 174

1879 168

1923 170

1827 230

1941 228

1843 289

1881 282

1923 290

1833 341

1938 341

1847 393

1885 386

1921 392

1841 434

1929 433

1869 484

1887 485

1905 482

1825 541

1886 572

1957 541

ID=Expansa_A1

LM=24

2047 114

2110 89

2170 109

2070 182

2111 180

2155 181

2049 254

2178 250

2066 316

2114 312

2158 317

2059 373

2170 370

2077 425

2115 424

2153 422

2073 465

2161 465

2102 509

2115 513

2133 510

2055 548

2115 570

2173 548

ID=Expansa_A2

LM=24

2270 113

2343 89

2417 109

2298 178

2349 177

2403 181

2286 245

2418 246

2309 306

2351 297

2399 305

2291 362

2411 360

2302 418

2351 408

2391 416

2299 456

2398 453

2327 501

2353 500

2370 498

2263 554

2358 572

2442 542

ID=Expansa_A3

LM=24

96 734

164 714

230 732

118 798

164 795

212 798

77 864

252 860

100 926

169 910

234 927

78 992

256 984

112 1043

168 1027

225 1038

109 1088

232 1091

152 1131

173 1132

193 1130

109 1172

174 1200

234 1172

ID=Lewyana_H1

LM=24

362 742

428 718

497 739

374 802

429 799

486 800

332 874

520 859

354 935

430 914

504 932

338 996

526 998

366 1056

437 1036

501 1052

369 1100

506 1099

413 1144

436 1144

460 1142

378 1183

441 1203

502 1182

ID=Lewyana_H2

LM=24

642 732

690 716

748 742

652 806

689 806

732 808

610 884

773 888

636 935

684 922

729 939

622 1003

744 1000

642 1052

684 1038

722 1052

637 1104

733 1102

660 1135

677 1138

693 1138

616 1178

674 1206

736 1183

ID=Lewyana_J

LM=24

861 735

924 714

989 736

882 811

925 806

970 811

858 878

998 875

880 935

929 926

977 932

868 996

997 988

886 1048

934 1038

980 1042

881 1094

984 1087

912 1132

932 1134

950 1134

868 1180

930 1203

992 1172

ID=Lewyana_A1

LM=24

1093 730

1152 715

1212 734

1109 796

1150 796

1194 799

1086 872

1216 868

1108 924

1152 908

1197 922

1088 983

1216 980

1110 1034

1150 1023

1189 1032

1102 1079

1201 1074

1132 1127

1148 1130

1168 1128

1081 1171

1145 1200

1210 1172

ID=Lewyana_A2

LM=24

1361 749

1429 714

1498 742

1378 798

1430 785

1482 794

1337 869

1529 862

1363 924

1430 909

1497 920

1338 985

1529 978

1374 1044

1437 1036

1498 1040

1358 1093

1518 1090

1409 1141

1441 1137

1471 1140

1366 1177

1447 1198

1518 1169

ID=Unifilis_H1

LM=24

1613 733

1674 716

1743 728

1629 804

1677 801

1731 800

1590 868

1774 862

1618 922

1679 909

1749 918

1594 990

1775 981

1630 1044

1686 1036

1741 1040

1609 1094

1762 1090

1663 1138

1687 1136

1710 1137

1618 1169

1691 1200

1754 1172

ID=Unifilis_H2

LM=24

1834 738

1897 716

1962 733

1853 800

1897 790

1938 797

1823 861

1971 856

1857 925

1902 921

1943 921

1826 988

1973 981

1853 1044

1902 1034

1951 1042

1837 1084

1965 1084

1879 1140

1902 1140

1926 1138

1838 1164

1906 1194

1971 1162

ID=Unifilis_J

LM=24

2065 734

2129 714

2185 730

2086 801

2129 804

2175 800

2065 858

2199 854

2091 924

2133 922

2181 924

2066 986

2201 982

2087 1050

2134 1046

2182 1048

2067 1089

2191 1088

2110 1138

2133 1141

2153 1137

2055 1166

2127 1200

2197 1164

ID=Unifilis_A1

LM=24

2279 728

2338 716

2401 722

2298 821

2346 809

2401 818

2285 873

2415 868

2309 932

2357 932

2401 926

2291 992

2414 984

2310 1042

2354 1040

2399 1038

2301 1084

2407 1080

2334 1136

2357 1138

2374 1136

2271 1177

2354 1205

2429 1166

ID=Unifilis_H2

LM=24

141 1366

194 1345

246 1368

152 1416

189 1414

226 1416

113 1486

264 1488

136 1541

184 1537

233 1542

116 1585

250 1584

146 1649

184 1652

226 1646

133 1680

237 1677

160 1724

184 1724

205 1722

122 1765

182 1793

245 1766

ID=Sextuberculata_H

LM=24

344 1369

418 1345

496 1366

384 1438

418 1436

458 1436

349 1508

498 1505

380 1562

426 1561

477 1560

366 1604

488 1602

386 1650

429 1664

472 1650

368 1686

486 1686

408 1729

430 1730

450 1726

364 1772

428 1796

494 1770

ID=Sextuberculata_J1

LM=24

580 1373

634 1345

693 1368

598 1417

633 1412

676 1413

573 1478

713 1468

594 1540

642 1537

693 1536

581 1586

714 1578

610 1644

654 1648

692 1641

598 1677

702 1673

634 1730

653 1729

673 1728

602 1764

658 1788

710 1764

ID=Sextuberculata_J2

LM=24

797 1370

845 1348

901 1365

816 1426

853 1422

889 1421

797 1490

912 1474

816 1532

858 1525

898 1529

810 1588

910 1576

828 1630

864 1628

897 1628

817 1673

906 1668

845 1714

864 1716

880 1713

801 1757

861 1794

924 1761

ID=Sextuberculata_A

LM=24

1072 1351

1127 1337

1187 1352

1081 1429

1123 1425

1171 1429

1055 1489

1200 1489

1073 1552

1123 1543

1175 1552

1051 1607

1193 1604

1075 1657

1123 1655

1168 1656

1071 1696

1175 1695

1103 1741

1120 1741

1139 1740

1068 1761

1123 1784

1176 1759

ID=Vogli_J

LM=24

1284 1356

1339 1343

1393 1351

1304 1432

1344 1423

1385 1427

1275 1492

1419 1483

1299 1541

1349 1531

1404 1535

1287 1592

1419 1584

1309 1640

1356 1627

1397 1631

1301 1681

1407 1673

1335 1721

1353 1720

1372 1719

1292 1759

1355 1784

1412 1752

ID=Vogli_A1

LM=24

1515 1360

1576 1340

1628 1357

1543 1443

1577 1449

1607 1444

1512 1507

1639 1504

1535 1568

1576 1563

1616 1572

1509 1612

1635 1607

1528 1663

1572 1664

1615 1663

1516 1695

1623 1691

1541 1739

1569 1741

1595 1739

1501 1769

1571 1791

1628 1764

ID=Vogli_A2

LM=24

1852 1373

1924 1340

1986 1376

1873 1453

1910 1442

1948 1452

1862 1505

1954 1506

1877 1546

1902 1538

1937 1548

1864 1588

1948 1589

1877 1632

1906 1632

1936 1629

1868 1665

1946 1662

1894 1706

1909 1708

1922 1706

1854 1757

1908 1788

1970 1768

ID=Pritchardi

LM=24

140 1950

212 1919

285 1947

161 2027

210 2025

264 2029

113 2083

306 2086

140 2158

209 2150

282 2155

110 2213

313 2209

150 2277

209 2271

278 2273

130 2306

296 2307

178 2346

213 2350

244 2346

158 2381

217 2402

270 2379

ID=Madagascariensis_H

LM=24

413 1946

485 1921

566 1950

433 2021

485 2018

541 2023

398 2094

566 2093

424 2162

482 2150

542 2159

401 2217

558 2217

426 2282

480 2278

526 2282

410 2315

540 2317

446 2355

473 2355

500 2355

414 2375

470 2402

530 2378

ID=Madagascariensis_J

LM=24

637 1937

696 1915

749 1945

660 2007

694 1999

729 2010

637 2075

754 2071

648 2137

692 2127

738 2137

637 2199

749 2198

654 2265

692 2262

729 2265

642 2301

734 2297

665 2343

686 2346

706 2343

638 2369

682 2406

732 2370

ID=Madagascariensis_A

LM=24

882 1945

946 1917

1010 1943

886 2002

947 2013

1006 2001

840 2079

1062 2069

880 2139

951 2125

1019 2138

855 2203

1044 2197

894 2271

950 2253

1007 2263

884 2302

1023 2301

931 2351

956 2347

982 2349

902 2378

958 2405

1015 2375

ID=Peltocephalus_H

LM=24

1122 1938

1175 1921

1234 1941

1119 1997

1179 2017

1230 1993

1094 2051

1255 2050

1115 2119

1174 2118

1227 2117

1090 2170

1256 2167

1120 2234

1174 2227

1226 2230

1116 2277

1239 2265

1152 2337

1180 2341

1203 2337

1116 2370

1184 2406

1244 2362

ID=Peltocephalus_J

LM=24

1328 1943

1382 1918

1443 1934

1336 2002

1390 2015

1442 1994

1307 2063

1471 2058

1331 2126

1386 2121

1436 2121

1307 2181

1474 2171

1338 2239

1391 2238

1440 2238

1336 2286

1456 2278

1376 2342

1399 2345

1420 2341

1347 2381

1406 2402

1460 2378

ID=Peltocephalus_A1

LM=24

1555 1941

1603 1918

1654 1939

1556 2011

1612 2029

1660 2013

1530 2067

1687 2067

1552 2131

1615 2121

1670 2127

1542 2178

1680 2174

1566 2238

1618 2230

1666 2230

1559 2279

1672 2271

1584 2337

1614 2337

1642 2334

1544 2366

1615 2406

1675 2369

ID=Peltocephalus_A2

LM=24

1805 1932

1845 1912

1888 1927

1815 1992

1860 1997

1897 1994

1797 2053

1929 2048

1799 2103

1860 2103

1921 2105

1791 2155

1938 2155

1809 2205

1863 2198

1929 2207

1808 2253

1934 2253

1844 2307

1872 2308

1897 2307

1802 2383

1875 2402

1932 2387

ID=Stupendemys_J

LM=24

2039 1931

2091 1914

2140 1924

2056 2004

2092 2000

2130 2004

2041 2054

2140 2050

2053 2099

2096 2092

2136 2099

2052 2144

2144 2137

2062 2177

2100 2170

2137 2173

2063 2216

2143 2211

2084 2269

2105 2273

2123 2271

2027 2364

2108 2405

2171 2362

ID=Stupendemys_A1

LM=24

2309 1945

2348 1915

2385 1925

2315 1995

2357 1989

2393 1988

2299 2036

2402 2025

2308 2068

2355 2061

2397 2064

2294 2108

2412 2102

2302 2141

2358 2138

2412 2141

2299 2183

2406 2184

2328 2230

2361 2235

2389 2231

2271 2374

2351 2398

2433 2366

Skull snout

LM=16

24 306

82 349

35 430

7 456

61 576

522 569

544 543

425 380

485 269

664 255

407 222

551 143

525 129

356 174

330 226

262 321

ID=PelSMF

LM=16

148 306

178 356

152 444

145 464

222 582

668 641

686 588

520 386

568 272

784 252

492 206

690 101

669 88

452 164

428 230

361 328

ID=PelAMNH131886

LM=16

80 422

128 524

122 602

102 628

182 758

672 790

702 750

540 582

600 426

858 394

528 360

672 246

642 226

452 304

444 380

352 496

ID=PelCRI1343

LM=16

109 348

136 426

112 530

105 560

165 645

634 684

664 645

486 465

536 380

846 390

550 290

774 253

684 121

462 220

457 270

342 398

ID=PelCRI1344

LM=16

49 341

85 398

70 500

64 537

126 637

644 605

654 570

509 365

553 257

780 222

486 202

713 77

646 66

429 156

416 204

293 326

ID=PelCRI3344

LM=16

44 345

81 398

32 478

18 510

57 622

530 654

528 600

404 441

482 294

730 260

462 230

641 145

613 130

358 202

344 240

253 360

ID=PelNFWFL336

LM=16

37 354

86 428

41 534

26 545

76 666

652 768

676 738

485 501

593 336

852 341

498 269

829 149

766 118

430 210

404 273

284 408

ID=PelSNM257687

LM=16

20 356

66 441

33 593

29 638

96 776

698 820

756 778

560 498

642 308

946 258

566 249

974 77

901 56

474 186

465 260

330 428

ID=PelSNM257688

LM=16

38 289

132 361

52 456

44 484

102 562

494 637

517 601

396 396

436 301

660 246

378 241

548 157

477 121

313 162

302 228

228 352

ID=MadaMNH

LM=16

118 330

190 402

118 512

98 542

148 654

682 710

696 648

588 400

624 320

878 256

574 248

758 150

740 118

468 206

446 252

306 392

ID=MadaNMW140

LM=16

90 366

182 430

102 536

88 582

140 682

658 744

692 648

554 472

594 370

852 298

536 276

748 206

718 168

470 214

434 268

286 414

ID=MadaNMW146

LM=16

41 218

118 293

29 370

9 388

45 466

432 529

468 502

384 340

454 241

604 234

410 172

558 133

528 93

317 125

321 161

188 294

ID=MadaSMF

LM=16

136 414

168 452

120 530

92 592

142 670

612 786

652 600

566 472

628 354

780 294

610 226

702 194

652 122

524 156

504 194

264 462

ID=EryCRI8207

LM=16

154 292

152 322

84 346

61 400

53 472

497 652

516 484

457 338

552 216

706 204

554 162

649 148

604 106

502 116

476 142

249 300

ID=EryCRI8208

LM=16

93 322

109 360

66 401

33 517

85 597

508 628

509 428

340 288

384 229

490 209

388 192

478 168

442 142

324 168

312 188

160 324

ID=ExpICN

LM=16

158 396

170 432

112 496

86 542

66 600

576 834

588 670

404 410

462 298

604 248

446 254

558 198

508 180

374 244

336 296

238 414

ID=ExpNMW137

LM=16

75 280

128 285

79 401

44 420

121 502

590 479

588 347

479 241

517 158

648 142

500 121

614 103

559 68

425 94

313 144

188 239

ID=LewICN1715

LM=16

89 258

110 282

91 310

72 379

119 425

379 457

394 334

329 255

363 204

453 190

369 162

433 152

413 126

350 129

299 150

170 231

ID=LewICN5769

LM=16

117 217

129 236

110 265

91 319

155 402

400 435

412 282

315 225

359 165

446 136

355 128

437 99

417 82

337 90

315 110

193 219

ID=LewICN7068

LM=16

131 186

142 201

122 223

87 292

183 389

393 400

420 258

360 223

414 159

496 142

411 131

486 115

474 100

374 105

324 132

223 172

ID=LewICN7653

LM=16

132 186

145 195

103 258

91 293

125 352

391 401

405 301

335 247

409 173

480 154

411 133

474 118

463 102

372 107

309 130

221 175

ID=LewMNHN

LM=16

154 264

174 279

137 334

104 410

162 489

536 519

482 403

325 269

380 208

508 178

430 154

530 133

500 102

405 119

345 154

222 241

ID=SexAMNH111069

LM=16

93 330

121 346

96 394

53 496

162 602

582 630

533 502

349 313

434 202

610 177

453 144

589 134

550 89

409 112

313 166

186 302

ID=SexAMNH111070

LM=16

88 352

116 372

93 416

22 524

110 642

556 688

500 581

306 406

458 233

542 210

458 206

532 189

546 140

424 156

365 190

182 320

ID=SexMNHN

LM=16

50 175

57 199

29 250

5 313

47 382

333 386

376 299

206 220

233 149

344 127

254 104

321 100

308 66

236 74

183 101

108 156

ID=SexMNHN

LM=16

105 257

116 270

62 333

20 429

37 510

420 509

428 410

273 277

362 174

482 158

385 142

478 137

464 117

346 121

252 161

166 233

ID=UniAMNH97118

LM=16

120 245

130 280

64 417

58 464

106 521

536 565

549 462

364 312

445 205

586 188

457 136

564 140

549 97

410 90

349 118

186 236

ID=UniCRI11120

LM=16

116 238

124 252

71 343

62 370

123 462

396 467

426 392

281 299

351 219

503 200

354 165

478 157

461 121

351 118

253 154

168 243

ID=UniMNHN

LM=16

80 205

91 228

49 315

38 330

61 380

325 395

355 338

249 254

292 179

383 167

307 132

374 118

357 80

261 91

218 119

123 192

ID=UniMTKD43353

LM=16

54 357

64 409

26 498

12 550

74 660

530 677

544 590

334 408

421 262

576 202

400 180

526 146

505 73

362 117

312 157

136 345

ID=VogUF39060

LM=16

116 336

137 357

68 509

53 557

101 637

589 700

606 601

388 424

496 270

676 198

488 178

629 156

614 118

464 122

373 166

213 309

ID=VogUF39100

LM=16

82 233

122 310

127 337

112 351

169 444

426 446

501 392

377 283

411 208

564 226

385 179

518 119

525 103

312 94

313 206

297 259

ID=Victo

LM=16

178 256

218 270

136 460

124 536

244 694

686 718

704 596

434 346

542 244

796 234

522 216

628 130

594 134

470 158

432 232

344 278

ID=CaniTata

LM=16

29 115

51 143

19 236

21 268

86 379

253 338

250 279

129 167

158 121

295 115

172 89

242 62

244 50

151 62

142 85

100 143

ID=CaniBra
