# Supplementary material for: New insights on the anatomy and ontogeny of the largest extinct freshwater turtles
Source: Heliyon. 2021 Dec 27;7(12):e08591. doi: 10.1016/j.heliyon.2021.e08591 (PMC8717240; doi:10.1016/j.heliyon.2021.e08591)
Supplement: Supplementary_FileS3.docx [file mmc3.docx]

**Supplementary File S3 for**

**New insights on the anatomy and ontogeny of the largest extinct freshwater turtles**

Edwin-Alberto. Cadena^1,2,*^, Andrés Link^3^, Siobhán B. Cooke^4^, Laura K. Stroik^5^, Andrés F. Vanegas^6^, Melissa Tallman^5^

^1^Universidad del Rosario, Facultad de Ciencias Naturales, Grupo de Investigación Paleontología Neotropical Tradicional y Molecular (PaleoNeo), Bogotá, Colombia

^2^ Smithsonian Tropical Research Institute, Panamá, Republic of Panama

^3^ Departamento de Ciencias Biológicas, Universidad de Los Andes, Bogotá, Colombia

^4^ Center for Functional Anatomy and Evolution, Johns Hopkins University School of Medicine, Baltimore, MD, USA

^5^Department of Biomedical Sciences, Grand Valley State University, Allendale, MI, USA

^6^Museo de Historia Natural la Tatacoa, La Victoria, Huila, Colombia.

**^*^Correspondence:** e-mail: edwin.cadena@urosario.edu.co (E-A. C)

**File S3. Changes to the character-taxon matrix of Joyce et al (2021)**

*Changes to characters definition or states*

**Character 75.** Secondary palate. 0= absent; 1= present, medially opened; 2= present, formed by medially fused palatines. *Remarks.* A third state was added to clarify the condition in *Stupendemys geographica* and other podocnemidids with presence of this feature formed differently.

*Changes to characters coding*

*Caninemys tridentata*

Character 18. From “?” to “1”

Character 19. *Caninemys tridentata* from “1” to “0”, based on DNPM-MCT-1496-R

Character 21. From “0” to “?”

Character 24. From “?” to “1”

Character 26. From “0” to “1”

Character 29. From “1” to “0”

Character 33. From “?” to “1”

Character 47. From “0” to “1”

Character 48. From “0” to “1”

Character 57. From “0” to “1”

Character 71. From “?” to “0”

Character 72. From “?” to “1”

Character 89. From “1” to “0”

Character 96. From “?” to “2”

Character 103. From “?” to “1”

Character 108. From “?” to “0”

Character 117. From “1” to “0”

Character 120. From “?” to “0”

*Erymnochelys madagascariensis*

Character 25. From “?” to “0” based on SMF-33056

Character 29. From “1” to “0”

Character 57. From “0” to “0&1”

Character 60. From “1” to “0”

Character 74. From “0” to “0&1”

Character 209. From “0” to “0&1”

*Peltocephalus dumerilianus*

Character 25. From “?” to “0”, based on SMF-40169

Character 49. From “0” to “0&2”

Character 52. From “1” to “2”

Character 60. From “1” to “0”

Character 74. From “0” to “0&1”

Character 208. From “2” to “1”

*Podocnemis bassleri*

Character 65. From “?” to “0”

Character 66. From “?” to “1”

Character 67. From “?” to “0”

Character 68. From “?” to “1”

Character 69. From “?” to “0”

Character 70. From “?” to “0”

Character 71. From “?” to “0”

Character 125. From “?” to “0”

Character 126. From “?” to “0”

*Podocnemis erythrocephala*

Character 67. From “-” to “0”

Character 69. From “-” to “0”

Character 208. From “2” to “1”

*Podocnemis expansa*

Character 52. From “?” to “0&1”

Character 65. From “1” to “0”

Character 66. From “-” to “1”

Character 67. From “-” to “0”

Character 68. From “-” to “1”

Character 69. From “-” to “0”

Character 209. From “0” to “0&1”

*Podocnemis lewyana*

Character 2. From “?” to “0”

Character 7. From “?” to “0”

Character 13. From “?” to “0”

Character 14. From “?” to “1”

Character 18. From “?” to “1”

Character 21. From “?” to “0”

Character 33. From “?” to “1”

Character 34. From “?” to “0”

Character 35. From “?” to “0”

Character 37. From “?” to “0”

Character 38. From “?” to “0”

Character 39. From “?” to “0”

Character 42. From “?” to “0”

Character 43. From “?” to “0”

Character 44. From “?” to “1”

Character 51. From “?” to “0”

Character 52. From “?” to “0&1”

Character 53. From “?” to “0”

Character 56. From “?” to “1”

Character 57. From “?” to “0”

Character 61. From “?” to “0”

Character 62. From “?” to “0

Character 63. From “?” to “0”

Character 64. From “?” to “0”

Character 105. From “?” to “0”

Character 107. From “?” to “0”

Character 109. From “?” to “0”

Character 119. From “?” to “2”

Character 120. From “?” to “0”

Character 124. From “?” to “0”

Character 125. From “?” to “0”

Character 127. From “?” to “0”

Character 128. From “?” to “0”

Character 129. From “?” to “2”

Character 130. From “?” to “2”

Character 166. From “?” to “1”

Character 168. From “?” to “0”

Character 184. From “?” to “-”, suprapygal is absent in this taxon

Character 185. From “?” to “-", suprapygal is absent in this taxon

*Podocnemis unifilis*

Character 33. From “?” to “1”

Character 52. From “?” to “0&1”

Character 209. From “0” to “0&1”

*Podocnemis vogli*

Character 69. From “?” to “0”
