# Supplementary material for: New insights on the anatomy and ontogeny of the largest extinct freshwater turtles
Source: Heliyon. 2021 Dec 27;7(12):e08591. doi: 10.1016/j.heliyon.2021.e08591 (PMC8717240; doi:10.1016/j.heliyon.2021.e08591)
Supplement: Supplementary_FileS5.docx [file mmc5.docx]

**Supplementary File S5 for**

**New insights on the anatomy and ontogeny of the largest extinct freshwater turtles**

Edwin-Alberto. Cadena^1,2,*^, Andrés Link^3^, Siobhán B. Cooke^4^, Laura K. Stroik^5^, Andrés F. Vanegas^6^, Melissa Tallman^5^

^1^Universidad del Rosario, Facultad de Ciencias Naturales, Grupo de Investigación Paleontología Neotropical Tradicional y Molecular (PaleoNeo), Bogotá, Colombia

^2^ Smithsonian Tropical Research Institute, Panamá, Republic of Panama

^3^ Departamento de Ciencias Biológicas, Universidad de Los Andes, Bogotá, Colombia

^4^ Center for Functional Anatomy and Evolution, Johns Hopkins University School of Medicine, Baltimore, MD, USA

^5^Department of Biomedical Sciences, Grand Valley State University, Allendale, MI, USA

^6^Museo de Historia Natural la Tatacoa, La Victoria, Huila, Colombia.

**^*^Correspondence:** e-mail: edwin.cadena@urosario.edu.co (E-A. C)

**File S5. Details of thoracic vertebra 1**

**
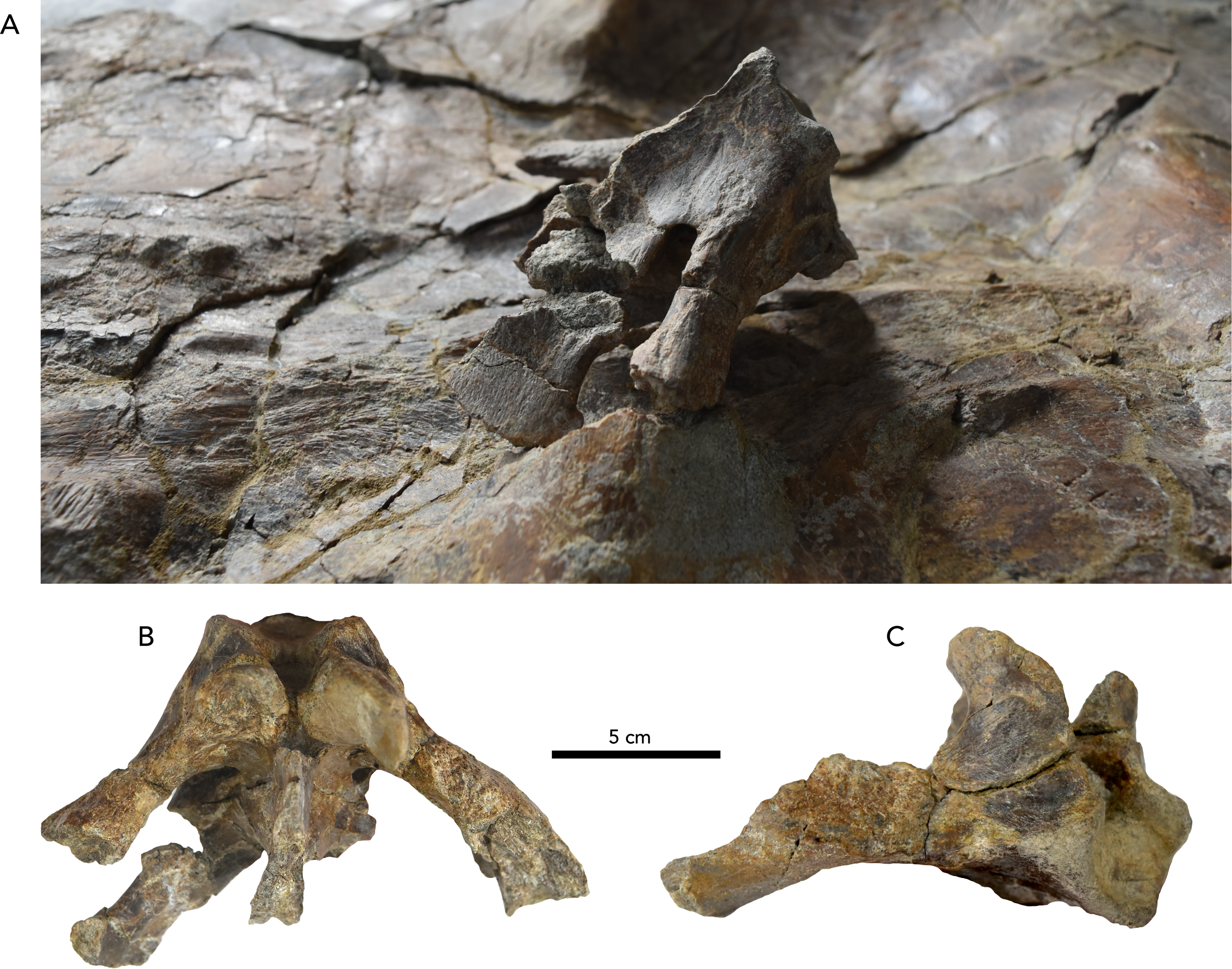
**

Details of the thoracic 1 of *Stupendemys geographica* VPPLT-1719 specimen

(A) Thoracic 1 vertebra articulated to the carapace in right ventrolateral view

(B) Thoracic 1 vertebra isolated in dorsal view

(C) Thoracic 1 vertebra isolated in right dorsolateral view
